# In Vitro Effects of Anaesthetic Compounds on a Panel of Solid Tumour Cell Lines—An Exploratory Study

**DOI:** 10.1111/jcmm.71307

**Published:** 2026-09-21

**Authors:** Olga Grajdieru, Alexandru Cristian Sabo, Adrian Bogdan Tigu, Andrei Ivancuta, Cristian Silviu Moldovan, Andrada Uhl, Miruna Cristina Ungurenasu, Andrada‐Ioana Balmez, Ada Moisescu, Simina Pîrv, Madalina Nistor, Ximena‐Maria Muresan, Diana Cenariu, Ciprian Tomuleasa, Catalin Constantinescu

**Affiliations:** ^1^ Department of Anaesthesia and Intensive Care Iuliu Hatieganu University of Medicine and Pharmacy Cluj‐Napoca Romania; ^2^ Department of Chemistry and Biochemistry USAMV Cluj‐Napoca Cluj‐Napoca Romania; ^3^ Department of Personalized Medicine and Rare Diseases, MEDFUTURE—Institute for Biomedical Research “Iuliu Hatieganu” University of Medicine and Pharmacy Cluj‐Napoca Romania; ^4^ Department of Nanosciences, MEDFUTURE Institute for Biomedical Research Iuliu Hațieganu University of Medicine and Pharmacy Cluj‐Napoca Romania; ^5^ Department of Hematology Iuliu Hațieganu University of Medicine and Pharmacy Cluj‐Napoca Romania

**Keywords:** anaesthetic compounds, cell death, morphological and structural changes, pain control, tumour cells

## Abstract

Anaesthetic agents are essential for surgical procedures, yet their potential role on tumour cell biology remains incompletely understood. Emerging evidence suggests that these compounds can act both as protumor and antitumor agents, influencing cellular processes such as proliferation, migration and cell death mechanisms. Eight commonly used anaesthetic compounds were tested on five tumour cell lines—A549Luc2, HCT116Luc2, HUH7, MDAMB231Luc2 and SKMEL28Luc2. Cytotoxicity was assessed by MTT assay, while morphological changes were analysed using confocal fluorescence microscopy. Functional assays were used for wound healing, colony formation, flow cytometry for cell death and cell cycle and gene expression profiling was assessed by RT‐qPCR. Morphological evaluation revealed profound compound‐specific alterations, including mitochondrial fragmentation, nuclear condensation, osmotic swelling and cell structure alterations. Lidocaine and ropivacaine induced stress fibre formation and swelling, while tunnelling nanotubes were observed as signs of cellular stress. The structural changes are correlated with impaired migration, reduced colony formation and necrosis‐dominated cell death. Gene expression analysis showed heterogeneous changes of apoptotic and cell cycle regulators, with caspase upregulation and NF‐kB activation in specific cases. Anaesthetic agents exert pleiotropic, cell‐line‐dependent effects, primarily inhibiting tumour growth and motility in our cell models through structural destabilization and stress signalling. These findings underscore the need for advanced models to clarify clinical relevance and to confirm the best choice of anaesthesia technique tailored to the patient's specific cancer type.

## 1 Introduction

Cancer remains one of the leading causes of death worldwide. The history of cancer treatment reflects a progressive development depending on biological understanding. For much of the 20th century, the therapeutic options were limited to the three classic pillars: surgery, cytotoxic chemotherapy and radiation therapy. Approaches that were often effective but carried substantial systemic toxicity and limited specificity. The introduction of molecularly targeted therapies in the late 1990s, followed by the immune checkpoint inhibitors in the 2010s, fundamentally shifted the paradigm from organ‐centric towards precision medicine guided by the molecular characteristics of individual tumours. Nowadays, cancer treatment is best understood as a continuum of evolving strategies, each building upon the biological insights of its predecessors, and each revealing new layers of complexity in tumour‐host interactions [1, 2].

Despite the advancements in cancer treatment, the disease remains a major burden on healthcare systems worldwide. Surgical intervention is one of the most effective methods for eradicating cancer, with millions of patients requiring these procedures annually [3]. Even when surgery and oncological treatments are performed correctly, sometimes cancer can recur or metastasize, resulting in increased mortality [4]. NETosis is described as a process where neutrophils are releasing decondensed chromatin to form neutrophil extracellular traps (NETs), which can trap the tumour cells from the bloodstream and hide them from the immune system, becoming a source for cancer recurrence [5]. Various hypotheses have asked the question of whether and which perioperative factors can influence the risk of cancer recurrence. As anaesthesia is required for surgical procedures, one of the hypotheses examines whether anaesthesia‐related drugs and techniques can influence cancer‐related outcomes.

Anaesthesia requires multiple key elements in order to be complete, like: anxiolysis, amnesia, hypnosis, analgesia and muscle relaxation. No single drug fulfils all of these criteria so a combination of agents is typically administered to the patient, each of them with its own specific properties. Commonly used anaesthetic drugs in clinical practice are categorized into volatile anaesthetics, intravenous anaesthetics, opioids, muscle relaxants and local anaesthetics. These are routinely employed across surgical scenarios, often without understanding all their consequences on different cellular types [6]. Recent advances suggest that anaesthetic drugs may influence the biomolecular pathways involved in different physiological and pathophysiological cellular mechanisms, such as proliferation, angiogenesis or cell death [7, 8].

The mentioned anaesthetic compounds have demonstrated various effects on tumour cell lines, in vitro, with implications for cancer biology and oncological patients. Volatile anaesthetics, such as isoflurane and sevoflurane, have shown potential pro‐proliferative effects and could induce chemoresistance in tumour cells, through upregulating HIF‐1α, leading to an enhanced epithelial‐to‐mesenchymal transition (EMT), angiogenesis and potential surviving metastatic tumour cells, effects observed at clinically relevant concentrations [9, 10]. Moreover, other studies suggest that sevoflurane, isoflurane or halothane demonstrated cytotoxic and antiproliferative effects on different cell lines in a cell line and time dependent manner [7, 11].

On the other hand, local anaesthetics have shown antitumor effect, inducing apoptosis, lowering proliferation rates and modulating critical cell signalling pathways, often being synergic with chemotherapeutic agents. Local anaesthetics like lidocaine, ropivacaine, bupivacaine and levobupivacaine had cytotoxic effects and inhibited tumour cell proliferation. There are various cell lines including triple negative breast cancer cells (TNBC) MDAMB231, triple positive brat cancer cells BT‐474, prostate—PC3 and LNCaP cells, bladder—HT1376 and TCCSUP cells, osteosarcoma, melanoma and even oral squamous cell carcinoma (OSCC). The compounds induced apoptosis, inhibited cell proliferation and promoted autophagy by producing mitochondrial dysfunction, caspase activation and downregulation of matrix metalloproteinases [12, 13, 14, 15, 16].

Neuromuscular blocking agents such as rocuronium bromide and succinylcholine (Suxamethonium Chloride) do not exhibit antitumor effects in vitro. In some TNBC models, rocuronium bromide was shown to promote invasion and growth, while succinylcholine had no effect on motility and growth, while in gastric cancer it stimulated cell growth [17, 18].

Propofol, an intravenous general anaesthetic agent, demonstrated dose‐dependent suppression of proliferation and migration on multiple tumour cell lines including lung cancer, colon cancer, breast, glioma and melanoma cells, promoting apoptosis and cell death and modulating critical pathways by inducing cell cycle arrest and reducing metastatic potential [19, 20, 21, 22, 23]. Same as propofol, ketamine inhibits tumour cell proliferation and migration, with demonstrated effects on various types of cancer cell lines including lung cancer, breast and colorectal cancer cells. It induces apoptosis and cell death, increases reactive oxygen species and suppresses cell cycle progression [24, 25, 26].

In some studies, opioids have shown stimulatory effects on tumour cells, suppressing immune surveillance and potentially promoting metastatic spread [27, 28]. For example, morphine and synthetic agonists can modulate tumour cell proliferation; others are showing dual effects, depending on their dose and exposure [29, 30]. Fentanyl, a widely used opioid for pain management in oncological patients, has also dual effects. While it can inhibit proliferation, invasion and migration ins different tumour cells, it can downregulate NFκB pathways, and suppress pro‐oncogenic transcription factors, it can also stimulate stemness and EMT in some breast cancer models, or it can stimulate angiogenesis in tumour‐associated endothelial cells at nanomolar concentrations [31, 32, 33]. There have been inconsistent results reported for pro‐tumour opioid effects in clinical settings; therefore, it is important to continue rigorous translational research on this topic so that in vitro data can be correlated with patient outcomes.

Noradrenaline, a naturally occurring catecholamine and a drug used as a vasoconstrictor in clinical practice, exerts heterogeneous effects on tumour cells in vitro, with outcomes that are highly dependent on tumour type, receptor subtype expression and cellular context. In several models, noradrenaline stimulated migration, invasion and proliferation, upregulating MMP2, MMP‐9 and VEGF genes, which promote the metastatic state of tumour cells in prostate cancer cells [34, 35]. Other studies showed that in pancreatic cancer cells and glioblastoma were inhibited by noradrenaline through increased cAMP signalling and MMP‐11 downregulation, while in breast cancer spheroid size was reduced [36, 37, 38, 39].

Based on these findings, there are inconsistent results that have no confirmation in clinical trials [28, 40] and justify further research in this area.

In search for a personalized anaesthetic technique tailored to the patient's specific cancer type, we aimed to evaluate the biological effects of commonly used anaesthetic agents on a panel of tumour cell lines, investigating the influence on cell proliferation, viability, cell cycle and changes on gene expression. The generated results will provide an overview of selecting tumour cell models which can be translated into in vivo experimental setups.

## 2 Materials and Methods

### 2.1 Cell Culture

A panel of 6 cell lines were used for cytotoxicity assay and based on their particularities, different functional assays and gene expression were performed. Lung adenocarcinoma cell line A549 Luc2+ (ATCC‐CCL‐185‐LUC2), colorectal cancer cell line HCT‐116 Luc2 (ATCC‐CCL‐247‐LUC2), Hepatocarcinoma cell line HUH‐7 (ABM‐T8973), melanoma cell line SKMEL28 Luc2 (ATCC‐HBT‐72– *in house* modified to express Luc2), Triple Negative Breast Cancer cell lines MDAMB231 Luc2 (ATCC‐HBT‐26—*in house* modified to express Luc2) and human fibroblast CCD1137Sk (ATCC‐CRL‐2703). The culture media and all supplements were purchased from Gibco (Gibco, Grand Island, MA, USA). RPMI 1640 supplemented with 10% Foetal Bovine Serum (FBS), 1% glutamine and 1% penicillin/streptomycin was used for A549 Luc2, HCT116 Luc2 and MDAMB231 Luc2 cells, MEM supplemented with 10% FBS, 1% glutamine and 1% penicillin/streptomycin was used for SKMEL28 Luc2, DMEM 4.5 g/L glucose supplemented with 10% FBS, 1% glutamine and 1% penicillin/streptomycin was used for HUH7 and IMDM supplemented with 10% FBS, 1% glutamine and 1% penicillin/streptomycin was used for CCD1137Sk. All cells were maintained in sterile conditions, at 37°C, 5% CO_2_ in humidified chamber. Bioinformatic annotation table for the malignant cell lines regarding their mutational status (Table 1).

**TABLE 1:** Mutational status of the cell lines used.

Cell line	Tumour type	p53 status	KRAS status	BRAF status	Other key features	Approximate doubling time	References
A549 Luc2	Lung adenocarcinoma	Wild‐type	Mutant (G12S)	Wild‐type	STK11/LKB1 mutant; CDKN2A homozygous deletion; high NF‐κB activity	~22 h	[41]
MDA‐MB‐231 Luc2	Triple‐negative breast cancer	Mutant (R280K, gain‐of‐function)	Wild‐type	Mutant (G464V)	Mesenchymal/claudin‐low subtype; high invasive capacity; Nrf2‐responsive	~28–38 h	[42]
HCT116 Luc2	Colorectal carcinoma	Wild‐type	Mutant (G13D)	Wild‐type	MSI‐high (MLH1 deficient); PIK3CA mutant (H1047R); intact apoptotic machinery	~18–21 h	[43]
SK‐MEL‐28 Luc2	Cutaneous melanoma	Mutant (L145R, non‐functional)	Wild‐type	Mutant (V600E)	High constitutive p53 protein accumulation; p53 transcriptionally inactive; CDKN2A deleted	~30–35 h	[44]
HUH7	Hepatocellular carcinoma	Mutant (Y220C, gain‐of‐function)	Wild‐type	Wild‐type	Mutant p53 with prolonged half‐life and nuclear accumulation; CTNNB1 (β‐catenin) pathway active	~24–30 h	[45]

### 2.2 Anaesthetic Compounds and MTT Assay

The effect of 8 anaesthetic compounds on different cell lines was studies by 3‐(4,5‐dimethylthiazol‐2‐yl)‐2,5‐diphenyltetrazolium bromide (MTT) assay. The names, stock solution concentration, concentration range and abbreviations are listed in Table 2.

**TABLE 2:** Anaesthetic compounds.

Compound name	Stoc concentration/mM	Interval tested (8 doses)	Abbreviation used
Noradrenaline	5.9 mM	590 μM−0.059 nM	P1
Lidocaine	42.7 mM	4270 μM−0.427 nM	P2
Rocuronium bromide	18.9 mM	1890 μM−0.189 nM	P3
Suxamethonium chloride	55.4 mM	5540 μM−0.554 nM	P4
Ropivacaine	36.5 mM	3650 μM−0.365 nM	P5
Fentanyl	0.148 mM	14.8 μM−0.001 nM	P6
Ketamine	210 mM	21,000 μM−2.1 nM	P7
Propofol	56.1 mM	5610 μM−0.561 nM	P8

The assay involves the formazan internalization into metabolic active cells, resulting in a purple colour formazan product whose absorbance at 570 nm is proportional with the number of metabolic active cells (further considered viable cells). The anaesthetic compounds were tested using a serial dilution, evaluating different concentrations of these compounds for 48 h incubation on six different cell lines—five tumour cell lines and one normal cell line. All cells were seeded into 96 well plates, at a density of 1 × 10^4^ cells per each well and incubated for 24 h to allow the cells to adhere. After 24 h, the treatment at various concentrations was added to each well. Additionally, each plate had an untreated control (representing 100% viability) and one group treated with 10% DMSO (representing the negative control—0% viability). After 48 h of incubation at 37°C, 5% CO_2_ and humidified atmosphere. Thereafter, the wells were washed with Phosphate Buffer Saline solution, and 100 μL of 1 mg/mL MTT Solution was added to each well on the plate and incubated for 4 h at 37°C, 5% CO_2_ and humidified atmosphere. The MTT solution was removed and 100 μL of DMSO was added to each well and the plates were read using a TECAN SPARK 10 M multiplate reader (TECAN, Männedorf, Switzerland). The experiment was repeated three times, and statistical analysis was performed using GraphPad Prism 8 software (GraphPad software, San Diego, CA, USA). Cell viabilities were expressed as percentages of control, and the data was analysed by one‐way ANOVA followed by Dunnett's multiple‐comparison test [46].

### 2.3 Morphological Evaluation by Fluorescence Microscopy

Cells were grown in chamber slides (5000 cells/well) control groups and treated groups with anaesthetic compounds at IC_25_. After the treatment, cells were washed with PBS and stained with CytoPainter Mitochondrial Staining Kit‐Red Fluorescence (Abcam, Cambridge, UK) for 1 h at 37°C. Thereafter, the cells were permeabilized using 0.5% Triton X and fixed with 4% Paraformaldehyde for 10 min at room temperature in dark. Thereafter, the cells were stained with Phalloidin‐FITC (Actinstain TM 488 Fluorescent Phalloidin, Cytoskeleton, Denver, CO, USA) and incubated at room temperature in darkness for 30 min. The cells were washed three times with PBS, and the nuclei were stained with 100 μL DAPI (Abcam, Cambridge, UK). The chamber slides were mounted in ProLong Gold Antifade mounting media and incubated at room temperature for 24 h. Cell images were obtained using an Olympus FLUOVIEW FV1200 laser scanning fluorescence confocal microscope. Image acquisition was performed using PLAPON60xOSC2 (1.4 NA) objective. The images were obtained using sequential mode (Three channels 405 nm, 488 nm and 543 nm excitation). Other settings for the image acquisition were determined by the software FV10‐ASW v.4.2, depending on the fluorescent dyes. Images were processed using Image J Software (National Institute of Health, Bethesda, MD, USA) [47].

### 2.4 Colony Assay

Equal number of cells (500 cells/well) were seeded in a 6 well plate, both for control groups and treated groups. The cells were incubated for 24 h to allow the cells to adhere, and then the anaesthetic compounds were added at an IC_25_ concentration, for 48 h. After 48 h, the culture media was replaced, and the plates were evaluated after 2 weeks. The culture media was removed, and 1 mL of ice‐cold pure methanol was added for 2 min, to fix the colonies. Thereafter, the methanol was removed, and 1 mL of violet crystals was added and incubated at room temperature for 15 min. The plates were washed with tap water and dried at room temperature for 24 h. After 24 h, the colonies were evaluated using the BioRad Chemidoc using the Coomassie blue settings. The wells were cropped and visually evaluated for the density of colonies [46].

### 2.5 Wound Healing Assay

The cells were seeded in 96‐well plates with a flat bottom for both the control and treatments. A total of 2.5 × 10^4^ cells were added to each well and incubated overnight, the wound was made using a pipette tip and the treatment at IC_25_ was added. The healing process was monitored periodically until the control's wounds had closed. The wound was examined using a Zeiss Axio Vert A1 inverted microscope (Zeiss, Jena, Germany) and the wound area was measured using ZEN software (Zeiss, Jena, Germany). For each condition, three replicates were measured. The wound closure was evaluated as percentages of the wound closure for every single well individually. The graphical representation was evaluated using GraphPad Prism 8 software [47, 48].

### 2.6 Apoptosis and Necrosis Evaluation

5 × 10^4^ cells were seeded into 6 well plates, for each group using triplicates (*n* = 3). After 24 h, when the cells were attached, the IC_25_ for each compound was added to the culture media and incubated for 24 h prior to Flow cytometry assessment. After the incubation, the cells were washed and detached using Trypsin/EDTA solution. The pellet was further washed and the cells stained with PoPRO1 + 7AAD (Thermo Scientific, Waltham, MA, USA). The staining procedure was for 15–30 min on ice, then the cells were immediately injected and evaluated using flow cytometer setup designed for PoPRO1 + 7AAD staining. The gating included Pacific Blue signal vs. PE cy5.5, and each group of cells had a negative control and an unstained sample for establishing the gating strategy.

The cell death was analysed using a BD FACS Canti II Flow cytometer, and the obtained data was processed with FACS DIVA v.6.0 software. The population of viable, Early apoptotic and Late apoptotic/necrotic cells was expressed as percentage of the total cell population [47, 49].

### 2.7 Cell Cycle

To assess the cell cycle changes, control samples and treated groups were tested. The cells were recovered from culture and fixed with ice‐cold 70% ethanol, and incubated for 1 h at 4°C. The cells were washed with PBS and incubated at room temperature for 15 min with RNAse A buffer (Thermo Scientific, Waltham, MA, USA). The propidium iodine (PI) solution of 2 mg/mL was further used for DNA staining. After 30 min of incubation at room temperature, in dark, the pellet was washed and resuspended in 500 μL of flow cytometry wash solution. The samples were analysed using a BD FACS Canti II Flow cytometer, and the obtained data was processed with FACS DIVA v.6.0 software [50].

### 2.8 Gene Expression

The RNA was extracted from cells (controls and treated groups) stored in 800 μL of Trireagent according to our previously published paper (Thermo Scientific, Waltham, MA, USA) [51]. The RNA was treated to remove the contamination with DNA, using TURBO DNAse Kit protocol (Thermo Scientific, Waltham, MA, USA) and the resulted RNA was used for cDNA synthesis—following the protocol for High‐Capacity cDNA reverse Transcription Kit (Thermo Scientific, Waltham, MA, USA) starting from the same amount of RNA. The RT PCR analysis was performed using Sso Advanced Universal SYBR green Supermix 2× from BioRad (BioRad, Hercules, CA, USA) at a final volume of 20 μL of mix with RNA. The reactions were analysed using the StepOnePlus RT PCR machine (Thermo Scientific, Walthman, MA, USA) in 96 well plates. The results were calculated as relative gene expression (2^−DDCT^) compared with the control samples (all groups had four replicates). The statistical analysis was performed using GraphPad Prism 8 software [52]. The primers used for gene expression analysis are detailed in Table 3.

**TABLE 3:** Primer sequences used for gene expression analysis.

*Gene*	Fwd.	Rv.
*Caspase 3*	TGTGAGGCGGTTGTAGAAGA	GGGCTCGCTAACTCCTCAC
*Caspase 8*	CCTGGGTGCGTCCACTTT	CAAGGTTCAAGTGACCAACTCAAG
*Caspase 9*	AAGCCCAAGCTCTTTTTCATC	ACTCGTCTTCAGGGGAAGTG
*NFR2*	ACACGGTCCACAGCTCATC	TGCCTCCAAAGTATGTCAATCA
*NF‐κB*	CACCGAAGCAATTGAAGTGA	GGCCTGAGAGGTGGTCTTC
*CDK2CYA*	AAAGCCAGAAACAAGTTGACG	GTACTGGGCACACCCTCAGT
*CDK1CYB*	TGGATCTGAAGAAATACTTGGATTCTA	CAATCCCCTGTAGGATTTGG
*CDK4CYD*	GTAGCAGTCGGTGGTACCTG	AGGCAGAGATTCGCTTGTGT
*GAPDH*	GTCTCCTCTGACTTCAACAGCG	ACCACCCTGTTGCTGTAGCCA

Abbreviations: Fwd., forward primer; Rv., reverse primer.

### 2.9 Western Blot Analysis

For the protein assessment, 3 × 10^5^ cells were seeded into a 6 well plate and treated with the IC25 dose of each compound for each cell line. After 24 h exposure to treatment, the cells were harvested and the pellet was homogenized in RIPA Lysis buffer supplemented with protease and phosphatase inhibitors. The lysate was centrifuged at 17,560 × g at 4°C for 10 min and the supernatants were collected. Total protein concentration was determined by Bradford method [53]. Protein samples were denatured in Laemmli buffer at 95°C for 5 min. Equal amount of protein (25 μg/sample) were separated by SDS‐PAGE on precast polyacrylamide gels and transferred to nitrocellulose membranes. The membranes were stained with Ponceau solution to check the transfer, then blocked for 1 h in 5% nonfat milk prepared in TBST. Further, the samples were incubated overnight with primary antibodies against cPARP (Invitrogen, rabbit anti‐human, 44‐6986), PARP (Novus, rabbit anti‐human, NBP‐09070), cCASP3 (Invitrogen, rabbit anti‐human, PA5‐114682), CASP3 (Invitrogen, mouse anti‐human, MA1‐16843) and against the normalizer B‐actin (R&D, mouse anti‐human, MAB8929). Signal detection was assessed after incubating with HRP‐conjugated antibody anti mouse (Merck, goat anti‐mouse, 12–349, batch 4,002,077) and antibody anti‐rabbit (Abcam, goat anti‐rabbit, AB97051) and incubation with substrate using ChemiDoc imaging system, and the band optical density was quantified with Fiji ImageJ software.

## 3 Results

### 3.1 Cytotoxicity Evaluation of the Anaesthetic Compounds on Tumour Cells

The effects on metabolic activity and cell viability were variate depending on the cell line and compounds that were tested. As depicted in Table 4, the IC_50_ doses for 48 h exposure to treatments were extremely different between the cell lines tested for each compound. CCD1137Sk, normal human fibroblast cell line, was used as a control to observe if the anaesthetic compound dose could inhibit normal cells. In this particular case only four out of eight compounds displayed inhibit effect—Noradrenaline (P1), Ropivacaine (P5), Ketamine (P7) and Propofol (P8) had inhibitory effect that could be used to calculate the IC_50_ values. For the Noradrenaline, the inhibitory dose was similar or lower than in tumour cell lines, while Ropivacaine, Ketamine and Propofol had higher IC_50_ values than in the case of tumour cell lines. In the case of Lidocaine (P2) the highest sensitivity was observed in HCT116 Luc2 cell line and HUH7 cell line, while MDAMB231 was the less sensitive tumour cell line. The Rocuronium Bromide had no inhibitory effect on HUH7 and MDAMB231 Luc2 cell lines, while SKMEL28Luc2 cells displayed high sensitivity to this compound. Suxamethonium Chloride had almost no inhibitory effect, except the 48 h treatment on HCT116 Luc2 cell line which was four times less sensitive to the compound than HUH7 cells. Fentanyl displayed no inhibitory effect on metabolic rate of all cell lines tested, as the IC_50_ could not be calculated. Ketamine effect was almost the same in all cases, with the normal cell line being the less sensitive, while in the case of tumour cell lines the IC_50_ was between 847 μM in the case of MDAMB231 Luc2 and 337 μM in the case of HCT116 Luc2. Propofol had variate effects, as MDAMB231 Luc2 and SKMEL28 Luc2 growth was not inhibited, while A549 Luc2, HCT116 Luc2 and HUH7 were sensitive to the compound. The variety in inhibitory effect justified the use of some doses even when the IC_50_ could not be calculated, for example in the case of Rocuronium Bromide (P3), Suxamethonium Chloride (P4) and Fentanyl (P6) compounds. The working doses are presented in Table 5, representing IC_25_ for the situations where it could be calculated. For those that IC_25_ could not be determined, the selected dose for further testing avoids physical–chemical changes in the media.

**TABLE 4:** The IC_50_ obtained for 48 h exposure to anaesthetic compounds.

	CCD1137 SK	SD	A549 Luc2	SD	HCT116 Luc2	SD	HUH7	SD	MDAMB231 Luc2	SD	SKMEL28 Luc2	SD
P1	147.50 μM	29.92	408.03 μM	11.90	9.57 μM	2.09	167.80 μM	19.63	26.07 μM	1.82	217.87 μM	9.45
P2	*n/a*	0.00	4106.67 μM	197.00	205.93 μM	49.12	305.90 μM	3.87	9557.67 μM	99.76	1426.00 μM	30.05
P3	*n/a*	0.00	6422.00 μM	579.83	1966.33 μM	1141.19	*n/a*	0.00	*n/a*	0.00	841.90 μM	37.43
P4	*n/a*	0.00	*n/a*	0.00	16098.67 μM	283.03	2131.67 μM	371.65	*n/a*	0.00	*n/a*	0.00
P5	2322.33 μM	52.54	1106.00 μM	89.02	413.17 μM	9.29	495.90 μM	11.13	1366.00 μM	373.40	647.53 μM	3.54
P6	*n/a*	0.00	*n/a*	0.00	*n/a*	0.00	*n/a*	0.00	*n/a*	0.00	*n/a*	0.00
P7	1504.33 μM	101.79	616.53 μM	34.87	337.30 μM	2.75	402.40 μM	2.25	847.93 μM	98.03	446.70 μM	28.20
P8	3499.67 μM	40.61	1478.00 μM	153.66	1340.67 μM	93.18	895.27 μM	239.33	9958.67 μM	954.16	3742.00 μM	172.20

*Note:* Compound without effect on cell metabolic activity.

Abbreviation: n/a, not applicable.

**TABLE 5:** The working dose for each compound for each cell line tested.

	A549 Luc2	HCT116 Luc2	HUH7	MDAMB231 Luc2	SKMEL28 Luc2
P1	351 μM	3 μM	77.5 μM	16.9 μM	106.7 μM
P2	1404.2 μM	55.6 μM	151.1 μM	2161.6 μM	519 μM
P3	1109.9 μM	29.3 μM	18.9 μM	18.9 μM	188.5 μM
P4	55.4 μM	5.98 μM	746.8 μM	55.4 μM	55.4 μM
P5	573.6 μM	346.7 μM	164.3 μM	340 μM	334.8 μM
P6	148 μM	148 μM	148 μM	148 μM	148 μM
P7	407.7 μM	197.9 μM	223.2 μM	562 μM	255.5 μM
P8	365.8 μM	481.6 μM	41.4 μM	3601.6 μM	98.6 μM

### 3.2 Morphological Changes Evaluated by Fluorescent Confocal Microscopy

**FIGURE 1:**
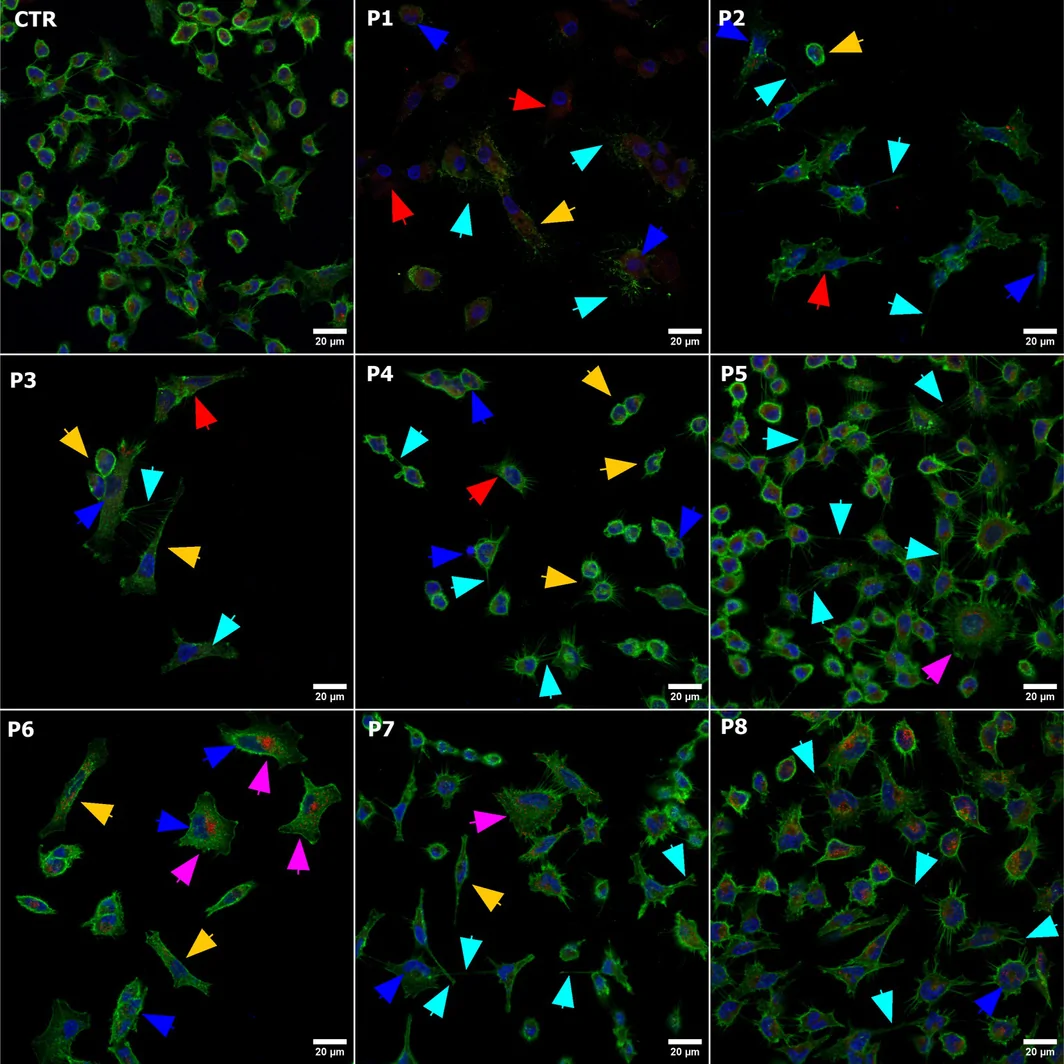
Anaesthetic compounds effect on A549 Luc2 lung adenocarcinoma cell line—Confocal evaluation of cell morphology. Red arrows—Mitochondrial loss/fragmentation or depolarization; Blue arrows—Nuclear morphology changes, condensation or fragmentation; Cyan arrows—F‐Actin filaments changes—Reorganization, stress fibres or filopodia, or tunnelling nanotubes; Magenta arrows—Cell volume changes, enlargement or swelling; Orange arrows—Cell shape changes, morphology changes or reorganization.

The morphological changes observed in lung cancer cell line A549 Luc2 are variate, from one compound to another. Noradrenalin (P1) induced changes in mitochondrial function, affected the nuclei, changed the cell shape, and in most cells the F‐actin filaments show fragmentation. Similar effects were observed in P2, P3 and P4. Cytoskeleton changes are observed in P5, where the cell population was not reduced, while stress fibre/filopodia can be observed, similar with the case of P7 and P8. In P5, P6 and P7, cell volume and osmotic stress can be observed. In P6, multiple cells changed their chape, and multiple cells display enlarged volume and nuclear modifications, as presented in Figure 1.

**FIGURE 2:**
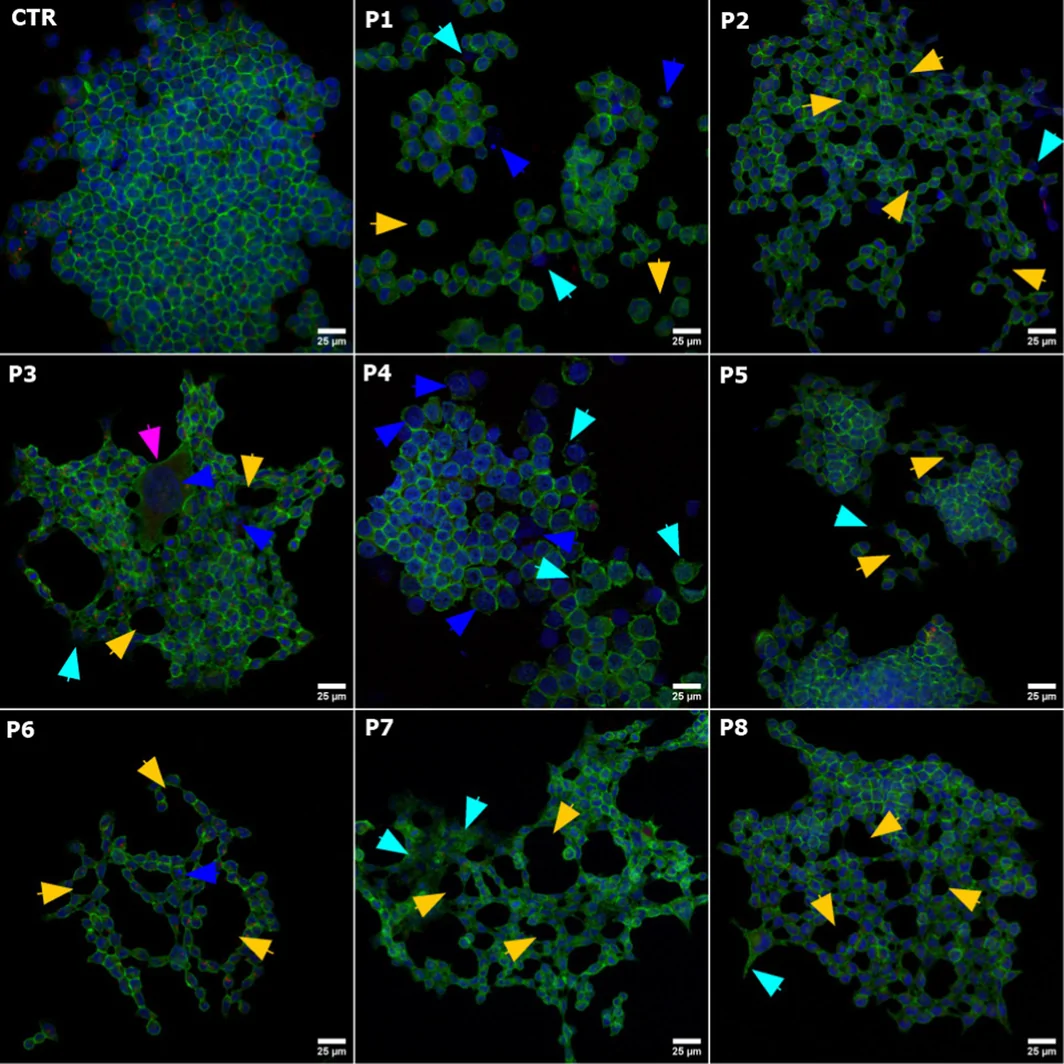
Anaesthetic compounds effect on HCT116 Luc2 colorectal cancer cell line—Confocal evaluation of cell morphology. Red arrows—Mitochondrial loss/fragmentation or depolarization; Blue arrows—Nuclear morphology changes, condensation or fragmentation; Cyan arrows—F‐Actin filaments changes—Reorganization, stress fibres or filopodia, or tunnelling nanotubes; Magenta arrows—Cell volume changes, enlargement or swelling; Orange arrows—Cell shape changes, morphology changes or reorganization.

HCT116 Luc2 cell population density was affected in all treated groups, in all groups excepting the P4, cells shape was changed, round shaped cells are visible (Figure 2). The nuclear modifications are observed in P1, P3, P4 and P6, with the most intense nuclear damage in P4 group. F‐actin filaments suffered changes in all groups, except P6, with many fragmented cytoskeleton regions and condensed filaments. In P3, one particular effect induced by the anaesthetic compound is the presence of cells with enlarged volume/swelling.

**FIGURE 3:**
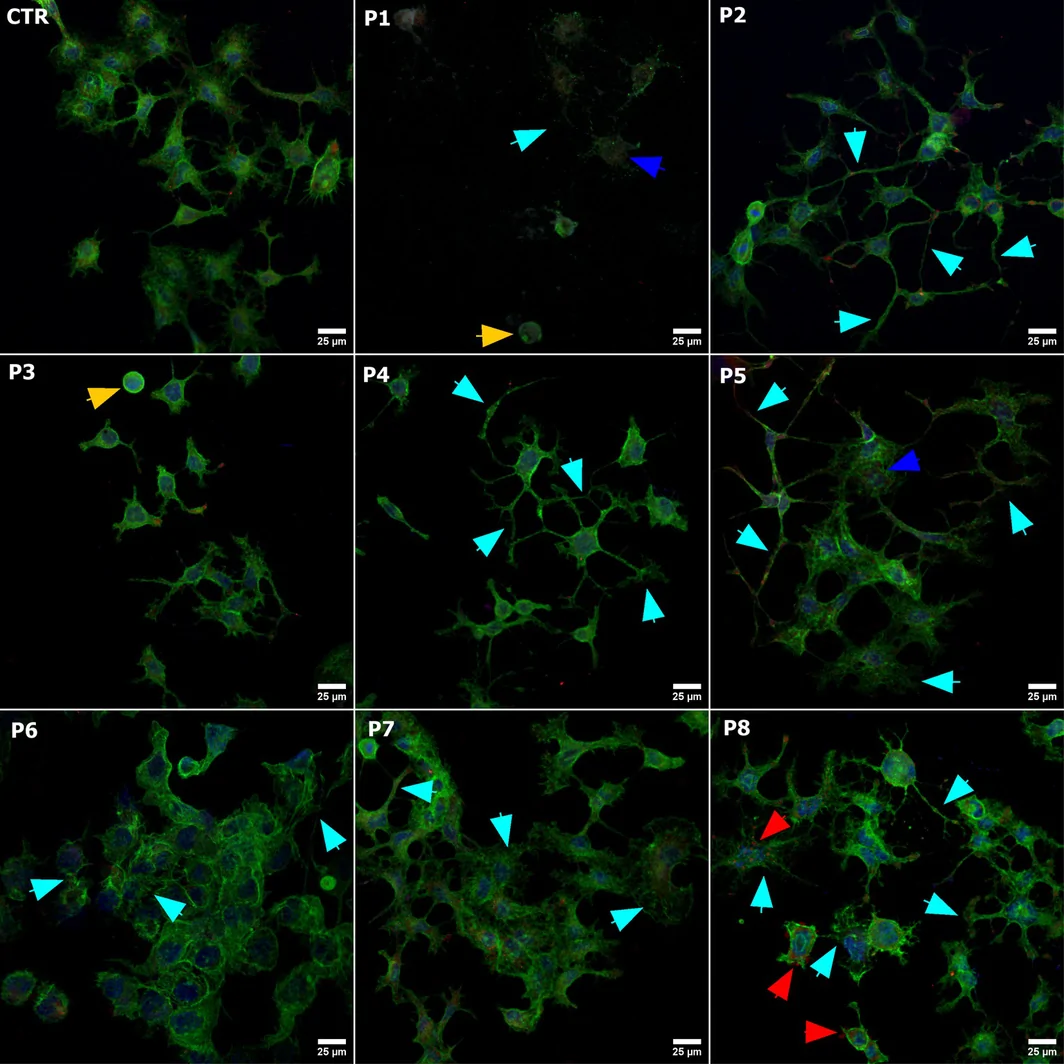
Anaesthetic compounds effect on HUH7 hepatic carcinoma cell line—Confocal evaluation of cell morphology. Red arrows—Mitochondrial loss/fragmentation or depolarization; Blue arrows—Nuclear morphology changes, condensation or fragmentation; Cyan arrows—F‐Actin filaments changes—Reorganization, stress fibres or filopodia, or tunnelling nanotubes; Magenta arrows—Cell volume changes, enlargement or swelling; Orange arrows—Cell shape changes, morphology changes or reorganization.

Hepatic cancer cells were affected by all anaesthetic compounds, mostly in terms of cytoskeleton reorganization and fragmentation. In P2, P4, P5 and P8, tunnelling nanotubes are observed. In P8, many mitochondrial fragmentations are observed, indicating cellular stress due to therapy. P1 and P3 reduced cell population and in both cases round shaped cells can be observed, while in other groups, round shaped cells are not present. The morphological evaluation is presented in Figure 3.

**FIGURE 4:**
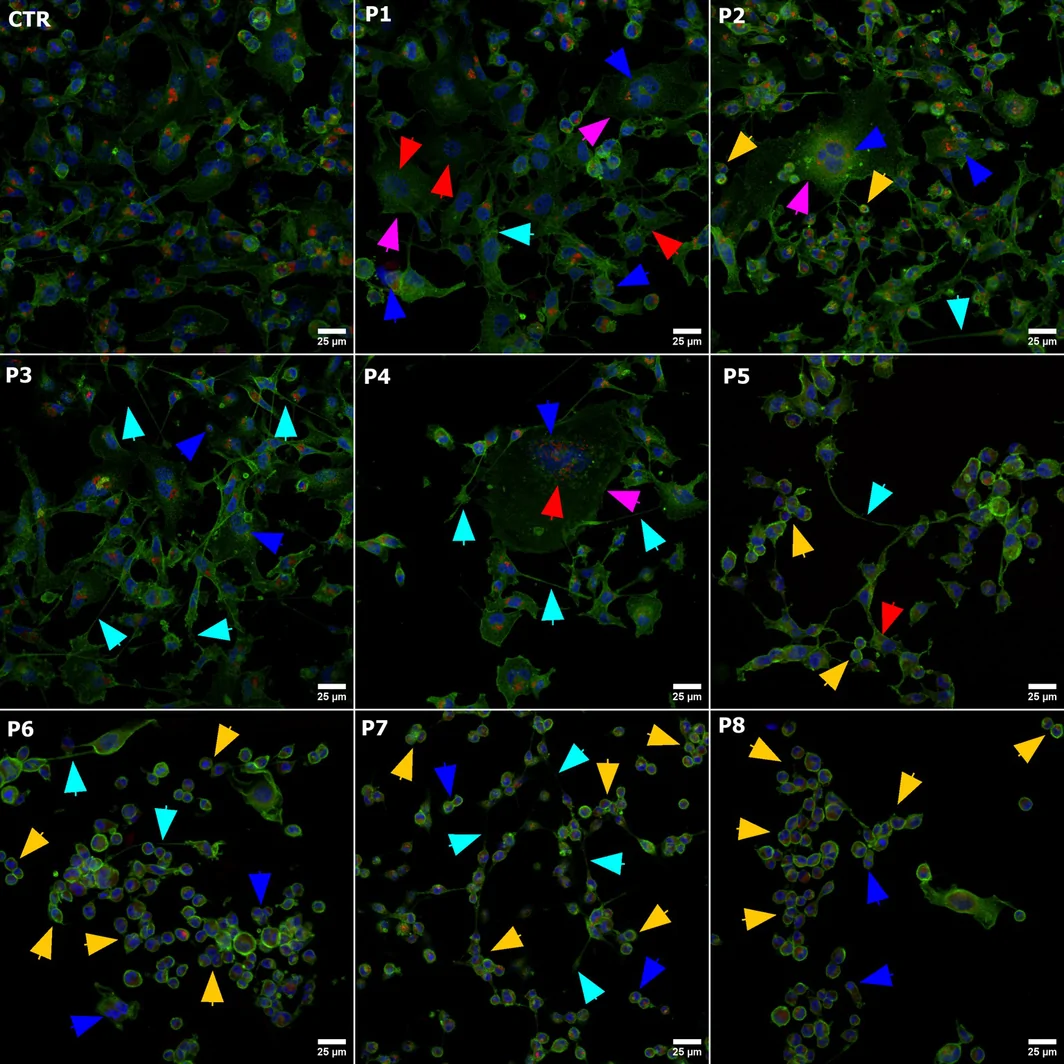
Anaesthetic compounds effect on MDAMB231 Luc2 Triple Negative Breast Cancer cell line—Confocal evaluation of cell morphology. Red arrows—Mitochondrial loss/fragmentation or depolarization; Blue arrows—Nuclear morphology changes, condensation or fragmentation; Cyan arrows—F‐Actin filaments changes—Reorganization, stress fibres or filopodia, or tunnelling nanotubes; Magenta arrows—cell volume changes, enlargement or swelling; Orange arrows—Cell shape changes, morphology changes or reorganization.

TNBC cells MDAMB231 Luc2 show changes in their cell populations, cytoskeleton reorganization, nuclear damage and changes in cell size, all changes being visible in a treatment dependent manner (Figure 4). F‐actin filaments were affected in all groups, except P8. Tunnelling nanotubes are observed in P2, P3, P4, P5, P6 and P7, highlighting the cellular stress. Cell body enlargement was observed in P1, P2 and P4, while round shaped cells are visible in P2, P5, P6, P7 and P8. Nuclear morphology was affected in all groups except P5, and mitochondrial fragmentation or depolarization were visible in P1, P4 and P5.

**FIGURE 5:**
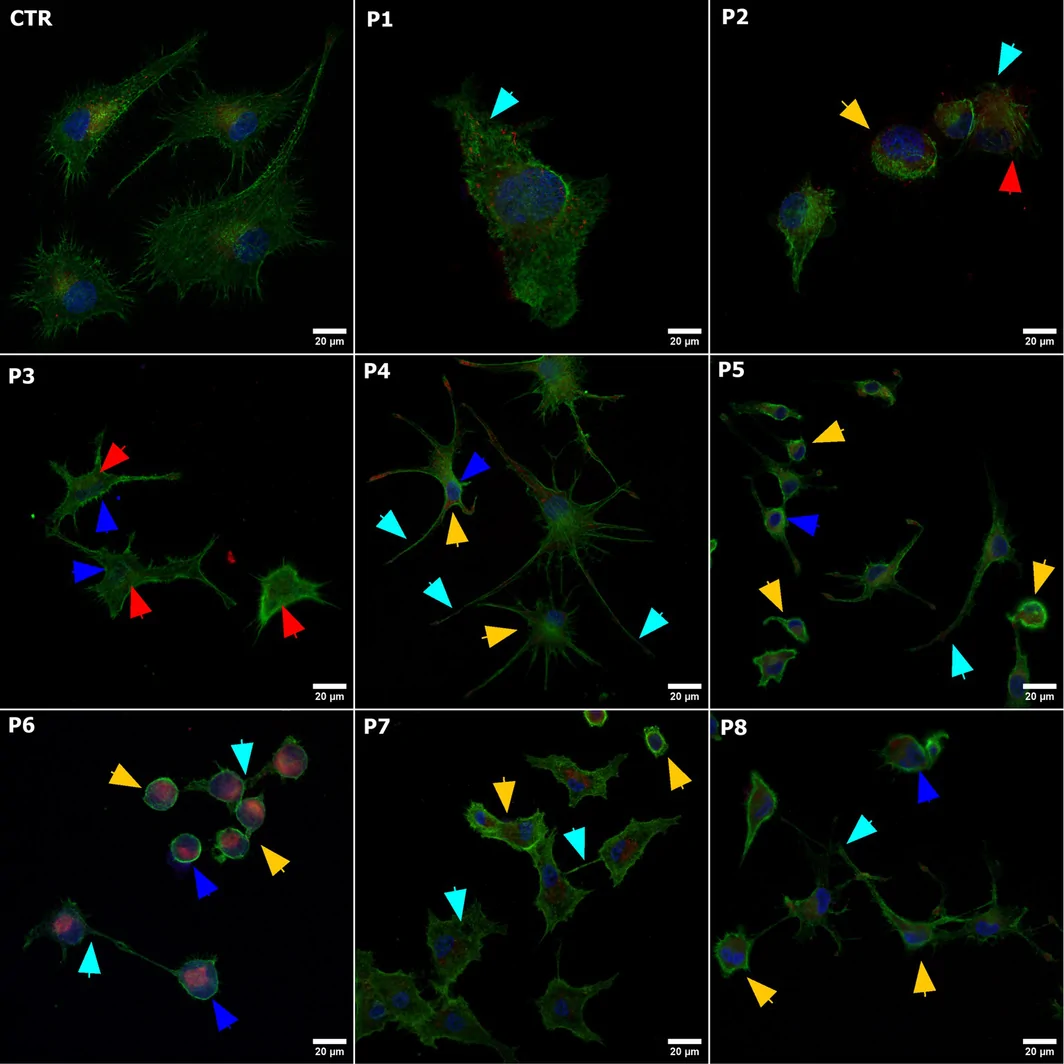
Anaesthetic compounds effect on SKMEL28 Luc2 Melanoma cell line—Confocal evaluation of cell morphology. Red arrows—Mitochondrial loss/fragmentation or depolarization; Blue arrows—Nuclear morphology changes, condensation or fragmentation; Cyan arrows—F‐Actin filaments changes—Reorganization, stress fibres or filopodia, or tunnelling nanotubes; Magenta arrows—cell volume changes, enlargement or swelling; Orange arrows—Cell shape changes, morphology changes or reorganization.

Melanoma cells SKMEL28 Luc2 have spectacular morphological features in the control group, with well‐defined actin filaments, healthy nuclei and visible mitochondrial networks, while all anaesthetic compounds induced changes in these cells, changing their morphology, reducing the cell size and in all cases except P1 and P3, round cells are visible. In P1, P2 and P7, actin filament fragmentation is visible, while in P4, P5, P6 and P8 multiple stress fibres are visible. P2 and P3 affected mitochondria networks, fragmenting or depolarizing the mitochondria, while nuclear damage is visible in P3, P4, P5, P6 and P8. The morphological changes are depicted in Figure 5.

All morphological changes are presented in Figures 1, 2, 3, 4, 5.

### 3.3 Wound Healing Assay

The IC_25_ dose for each compound was used for testing the inhibition of cell migration. Even if the doses that were selected are below the IC_50_, or in the case of compounds that had no effect on metabolic activity or viability, the doses were determined starting from the stock solution. The migration was inhibited in a cell line dependent manner. In the case of HUH7 the wound healing was not performed as the cells tend to grow in small clusters, and create networks of pseudopods, thus the wound could not be created using pipet tips.

A549 Luc2 migration was inhibited by all compounds. Wound closure above 50% was observed only P1, P3 and P6 treatments, while the rest of compounds were more effective. P4 had the most effective inhibitory effect on migration, with the wound closure of only 23%.

In the case of MDAMB231 Luc2 cells, P4 had no effect, while P1 and P5 kept the wound closure at almost 90%, compared to the other compounds that had significant inhibitory effect. P2 and P7 were the most effective in MDAMB231 Luc2 case, with a wound closure of < 30%.

Different situations are observed in the case of SKMEL28 Luc2, where the cell migration was not inhibited by any of the compounds, except P3 which completely inhibited cell migration.

HCT116 Luc2 showed sensitivity to all compounds used, which kept the wound healing between 61% and 85% closure.

**FIGURE 6:**
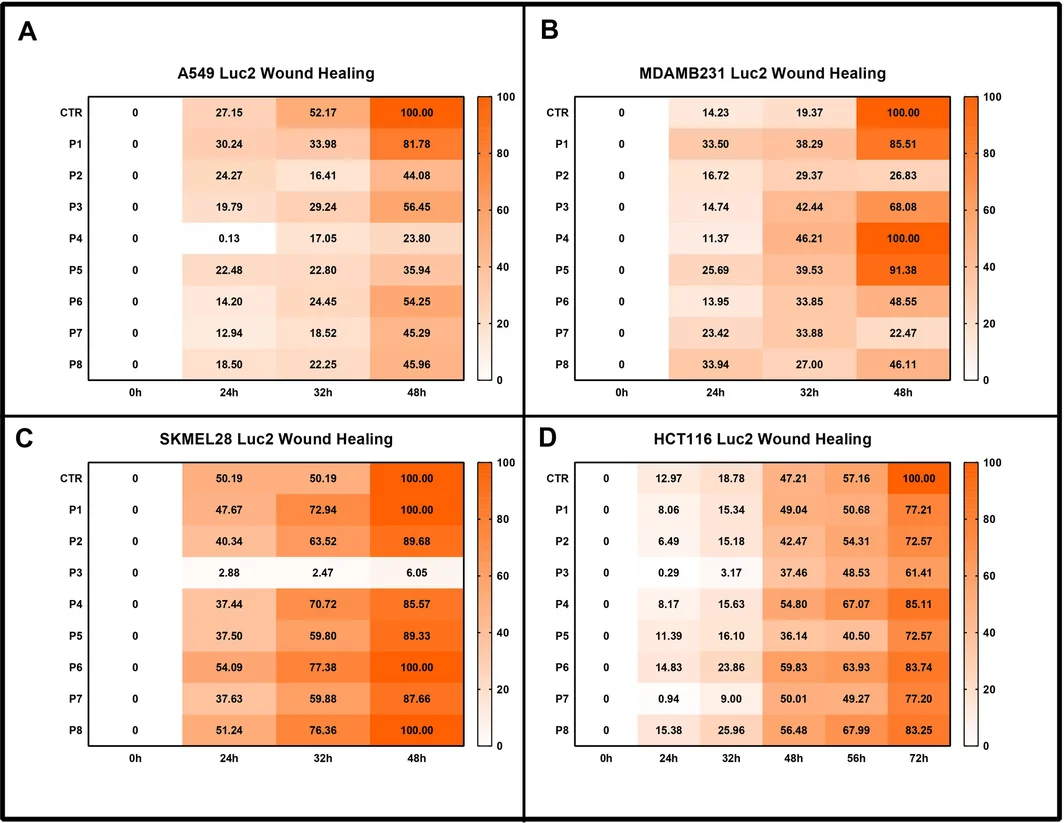
Wound healing evaluation on tumour cells. The wound closure was measured in percentage compared to the initial wound for each sample. Wound healing for HUH7 cell line was not possible due to cell line features.

The eight anaesthetic compounds displayed heterogenous effects on cell migration, depending on the cell line that was tested, as the tumour cells reacted differently to each compound (Figure 6). The percentage of wound closure is represented in different nuances of orange—light orange for less wound closure and dark orange for 100% closure. None of the treated compounds stimulated cell migration.

### 3.4 Colony Formation

Another tumour cell's key feature is represented by its ability to create new colonies from a single cell. The effect of each anaesthetic compound was tested on A549 Luc2, MDAMB231 Luc2, HUH7, SKMEL28 Luc2 and HCT 116 Luc2 cell lines and the results were highly heterogenous.

In the case of A549 Luc2 cells, the colony formation was significantly inhibited by P1, P3, P7 and P8, while P2, P4, P5 and P6 had no visible effect.

In the case of HCT116 Luc2, P6, P7 and P8 had significantly inhibited colony formation, while all other compounds had no effect on colony formation.

The TNBC cell line MDAMB231 Luc2 showed high sensitivity to P1, P2, P6, P7 and P8, in all cases the colony formation was significantly inhibited, while P3, P4 and P5 had no effect on colony formation.

The melanoma cell line, SKMEL28 Luc2, was the least sensitive to anaesthetic compounds in terms of colony formation, with P1 being the only treatment that inhibited colony formation.

Unfortunately, HUH7 cell line could not create colonies starting from 500 cells per well, thus, the evaluation was not possible as only few cells created small colonies that could not be counted.

**FIGURE 7:**
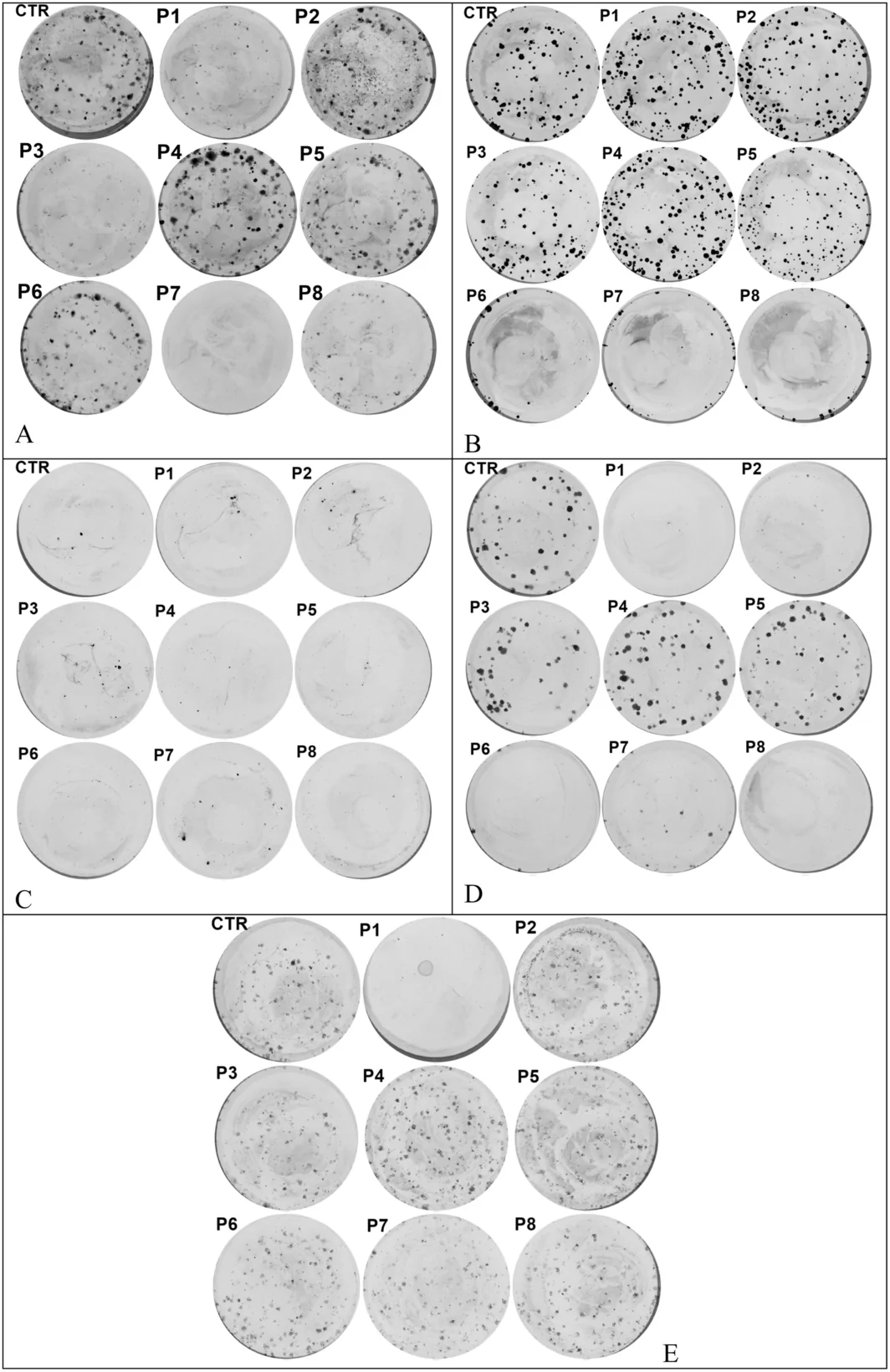
Colony formation assay. The tumour cell lines (A—A549 Luc2; B—HCT116 Luc2; C—HUH7; D—MDAMB231 Luc2; E—SKMEL28 Luc2) were evaluated after 48 h exposure to anaesthetic compounds.

Notably, these findings suggest anaesthetic compounds had different effects on the treated cell lines, with P7 and P8 showing inhibitory effects in all cases, even in SKMEL28 Luc2, where the effect was not significant, but still visible compared to the other compounds. The cell lines reacted differently depending on the compounds that were used, while no compounds showed stimulatory effects on colony formation, as presented in Figure 7.

### 3.5 Flow Cytometry Evaluation of Cell Cycle

The eight tested compounds had various effects at 48 h on the tumour cell lines.

**FIGURE 8:**
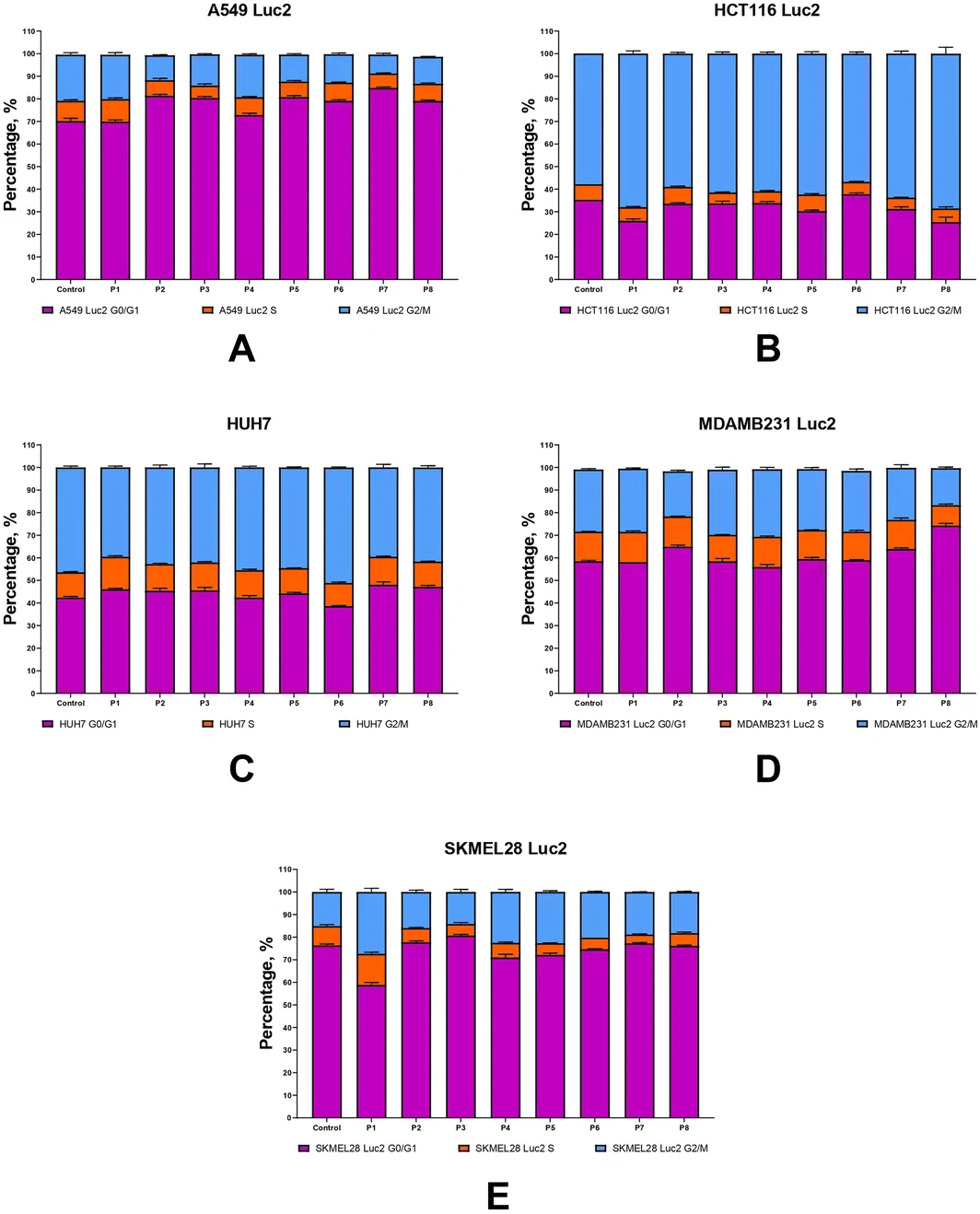
Flow cytometry evaluation of cell cycle. The tumour cell lines (A—A549 Luc2; B—HCT116 Luc2; C—HUH7; D—MDAMB231 Luc2; E—SKMEL28 Luc2) were evaluated after 48 h exposure to anaesthetic compounds.

In terms of cell cycle, the changes compared to the untreated group were observed in P2, P3, P5, P6, P7 and P8. In all cases the G0/G1 phase was increased, while both S phase and G2/M phase were reduced. The most pronounced effect of freezing cells in G0/G1 was observed in the group treated with P7 where almost 85% of the cells were frozen in G0/G1 compared to 70% in control group. No differences were observed in P1 and P4, where the anaesthetic compounds had no effect on the cell cycle. The changes observed in A549 Luc2 cells are depicted in Figure 8A.

The colon cancer cell line HCT116Luc2 treated with the anaesthetic compounds showed a shift in the cells into necrotic state in P7 and P8. The changes observed in the cell cycle of these cells were not significant, as excepting P1, P7 and P8, where the cell cycle arrest was observed in G2/M phase, lowering the G0/G1 phase, in all other groups (Figure 8B).

The effects observed on the cell cycle indicate that the treatments were not inducing changes in cell cycle state of the cells, except the P7 and P8 where the G1/G0 population increased compared to the control (Figure 8C).

TNBC cell line MDAMB231 Luc2 showed various effects after 48 h exposure to treatmentsThe cell death in MDAMB231 Luc2 cells can be related to the cell cycle changes, as only cells treated with P2 and P8 had significant changes in the cell cycle state. P2 induced a freeze in G0/G1, slightly increased than in control group, while P8 has a significant effect, freezing 70% of the cells in G0/G1, compared to 57% in control group (Figure 8D).

The cell cycle changes were variate, as P1 stimulated the freeze in S phase and G2/M phase, with the most significant change in G2/M phase out of all treatments. Increased G2/M population was observed in P4, P5, P6, P7 and P8 groups, where also the S phase was reduced compared to the control, increasing the G0/G1 (Figure 8E).

### 3.6 Cell Death Evaluation After 24 h Treatment

**FIGURE 9:**
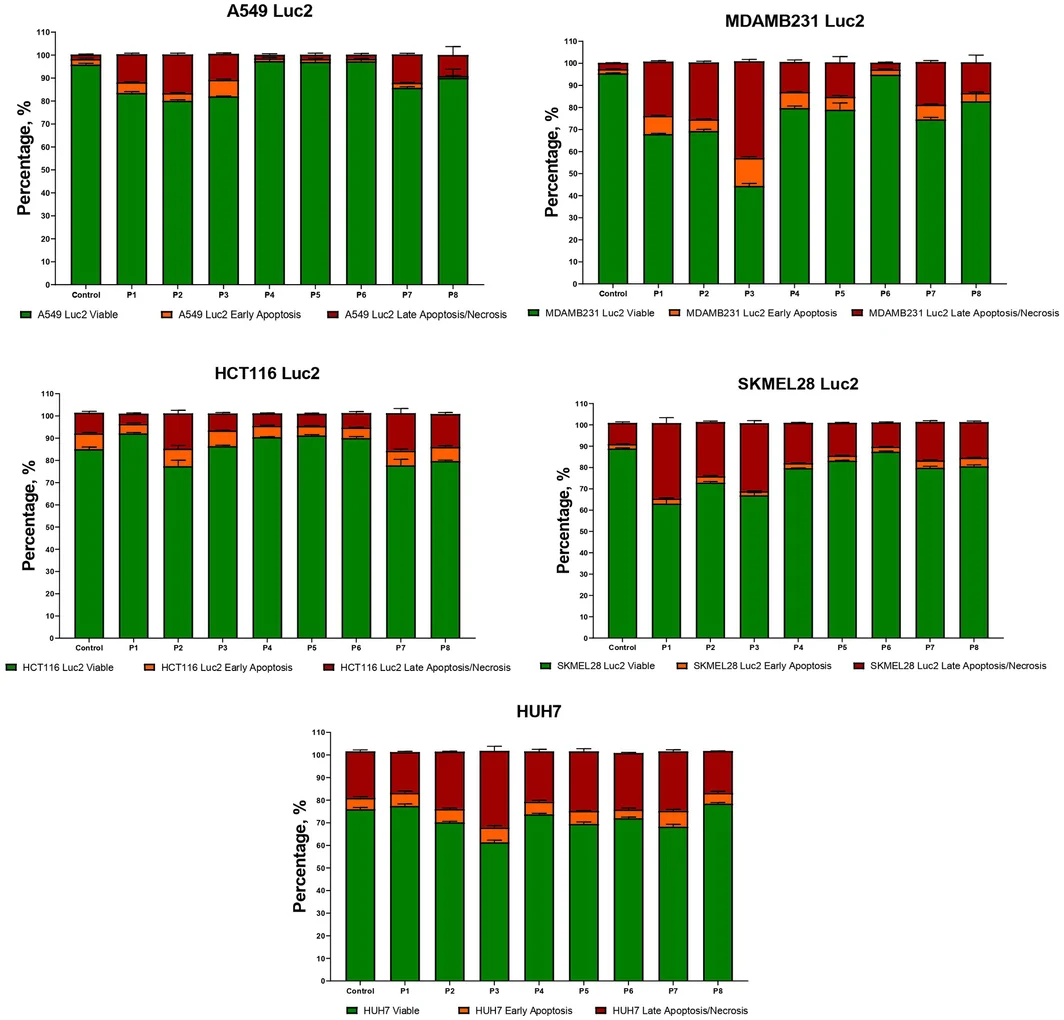
PoPRO1‐7AAD assessment for cell death.

Cell death evaluation highlighted various effect induced by the anaesthetic compounds to all five cell lines that were tested (Figure 9). In the case of A549 Luc2 cells, P4, P5 and P6 had no significant effect on cell viability, while the use of P1, P2 and P3 induced almost 15% cell death. P7 and P8 had also inhibit effect on cell viability, but with a less intense induced cell death, compared with the effects observed at P2 and P3.

In MDAMB231 Luc2 cells, all compounds except P6 induced significant cell death rates, with the most intense inhibitory rate observed in P3, where early apoptotic cell represent around 10% of the population and more than 35% were in late apoptosis. Other compounds had mild pro‐apoptotic effect, with less than 30% cell death rate.

HCT116 Luc2, the colorectal carcinoma cell line, is the less sensible to anaesthetic compounds, none of them induced more than 20% cell death, and the most intense effect was observed in P2, P7 and P8 groups.

SKMEL28 Luc2 cells were highly sensitive to P1 and P3, with around 35% apoptosis rate, while P2, which had significant effect on all other sell lines, showed only 20%–25% apoptosis rate. P6 is the least effective compound in term of cell death induction, while P4, P5, P7 and P8 had similar outputs with around 20% apoptotic rate.

Hepatocarcinoma cell line, HUH7, had a basal feature of 20% apoptotic cells, and the highest apoptotic cell percentage was observed in P3, where it reaches 30%, showing a 50% increase in apoptosis compared to the control. Other compounds showed no significant effect on HUH7 cell line, all maintaining the basal cellular phenotype.

### 3.7 Gene Expression Analysis

The gene expression evaluation included a panel of genes involved in cell death, response to oxidative stress, inflammation and genes controlling the cell cycle. In all cases, the molecular response at a gene expression level was variated, from one cell line to another.

**FIGURE 10:**
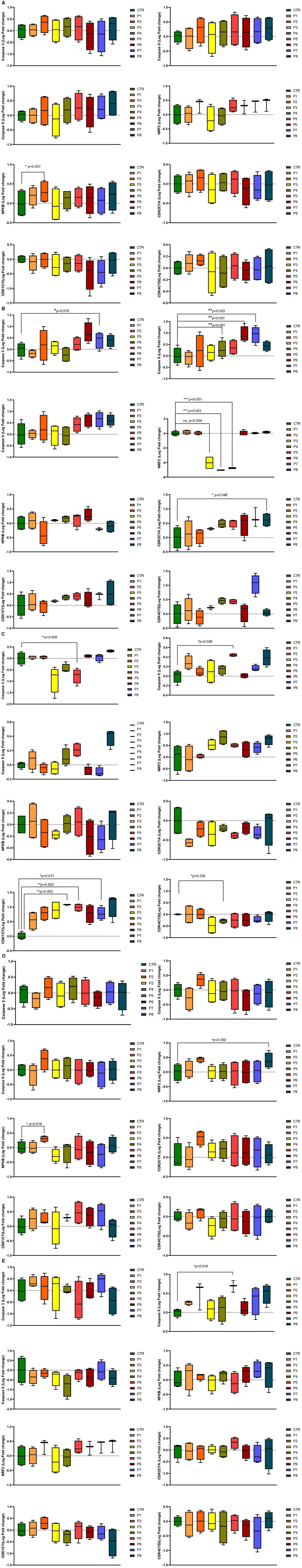
Gene expression evaluation. The tumour cell lines (A—A549 Luc2; B—HCT116 Luc2; C—HUH7; D—MDAMB231 Luc2; E—SKMEL28 Luc2) were evaluated after 48 h exposure to anaesthetic compounds.

In A549 Luc2 cells, the caspases had heterogenous gene expression, as *Caspase 3* had a trend to lower its expression, while *Caspase 8 and 9* showed a trend of an upregulation. However, none of them have significant changes compared to the control group. *NRF2* showed no significant changes after 48 h exposure to all treatments, while *NF‐κB* was significantly upregulated after exposure to P2 (*p* = 0.031). None of the cyclin genes had significant changes, however, the trend for groups treated with P6, P7 and P8 suggests a slight downregulation of *CDK1CY*. In the case of *CDK4CYD* the group treated with P2 showed an upregulation without any statistical significance compared to the control (Figure 10A).

In the HCT116 Luc2 cell line, the effect at gene expression level showed several changes that are statistically significant compared to the control group. *Caspase 3* expression was increased in groups treated with P6, P7 and P8, with the group treated with P7 having statistically significant upregulation (*p* = 0.016). Same as for *Caspase 3*, an upregulation of *Caspase 8* was observed in groups treated with P4, P5, P6, P7 and P8, with statistically significant upregulation after treatment with P5 (*p* = 0.001), P6 (*p* = 0.001) and P7 (*p* = 0.003). The trend was followed by *Caspase 9*, but without statistically significant changes. The effect on *NRF2* expression was observed only in groups treated with P3 (*p* = 0.054), P4 (*p* = 0.001) and P5 (*p* = 0.001), where all three compounds induced a downregulation of the gene. In the genes involved in cell cycle, no statistically significant changes were observed, except for the upregulation of *CDK2CYA* after exposure to P8 (=0.048). For the same gene, cells treated with P3, P4, P5, P6 and P7 had a trend indicating a slight upregulation, without statistical significance. Cell treated with P7 showed an upregulation of *CDK4CYD* without statistical significance, and the cell treated with P4, P5, P6, P7 and P8 followed a trend that suggested a slight upregulation of *CDK1CY* (Figure 10B).

The gene expression analysis for HUH7 cell line suggest a downregulation of *Caspase 3* in groups treated with P3, P4 and P5, with only P5 expressing statistically significant changes (*p* = 0.009), and the group treated with P8 had a slight upregulation of these genes. On the other hand, the gene expression for *Caspase 9* showed an upregulation pattern for groups treated with P5 and P8, and a downregulation pattern for groups treated with P6 and P7. *Caspase 8* was upregulated in groups treated with P1, P2, P4, P5, P7 and P8, with P5 showing statistically significant changes (*p* = 0.038). No significant changes were observed in *NRF2* and *NF‐κB* gene expression, while in the genes controlling cell cycle the differences were notable. All groups treated with anaesthetic compounds showed an upregulation of *CDK1CY*, with statistically significant differences in P4 (*p* = 0.003), P5 (*p* = 0.003) and P7 (0.017). On the other hand, *CDK4CYD* expression was downregulated in groups treated with P3, P4, P5, P6, P7 and P8, with statistically significant differences in P4 group (*p* = 0.035). In the case of *CDK2CYA*, all groups had a trend suggesting a downregulation of the gene, without statistically significant differences observed (Figure 10C).

The MDAMB231 Luc2 cell line showed the less significant differences in the gene expression of the analysed genes. Caspases showed no differences, while *NRF2* had a slight upregulation in groups treated with P2 and P8, with P8 showing statistically significant differences (*p* = 0.030). *NF‐κB* suffered significant changes in the group treated with P2 (0.018), being upregulated compared to the control. The genes controlling cell cycle had no significant changes (Figure 10D).

The effects observed in the SKMEL28 Luc2 cell line had no statistically significant differences compared to the control. The only exception is with P5 where *Caspase 8* had significant upregulation (*p* = 0.018).

In the case of *Caspase 8*, groups treaded with anaesthetic compounds had a trend showing a slight upregulation in all groups, while in the case of *Caspase 9*, the groups had a slight downregulation of the gene. None of the other genes had visible changes in terms of gene expression (Figure 10E).

### 3.8 Protein Evaluation by Western Blot

The mechanism of cell death controlled by PARP—a nuclear DNA repair enzyme, and Caspase 3—an inactive zymogen in the cytoplasm can highlight the apoptosis state in cells. PAPR signalling in A549 Luc2 cells was modulated mostly by P7 (ketamine), as total PARP was decreased and cPARP was maintained in all experimental groups. On the other hand, total CASP3 was decreased in P2 and P7, compared to the control, while cCASP3 was maintained low for all groups.

TNBC cell line MDAMB231 Luc2 had an increased level of total PARP while cPARP was lower than other groups. Slight increase in cPARP was observed in P4, P5 and P6, while in the case of CASP3, an increased level of total CASP3 is observed in P7 with cCASP3 still lower than the control for all treated groups.

In HCT116 cells, the detection of PARP was possible in the treated cells except the P2, P7 and P8, where all the proteins were barely expressed, indicating that the treatment might have an effect on protein integrity.

Melanoma cell line SKMEL28 had no significant changes observed in all experimental groups, as in all cases, the binding between antibody and target protein showed no PAPR or CASP3 detection. Indicating that this cell line might rely on other pathways for inducing cell death or survival.

**FIGURE 11:**
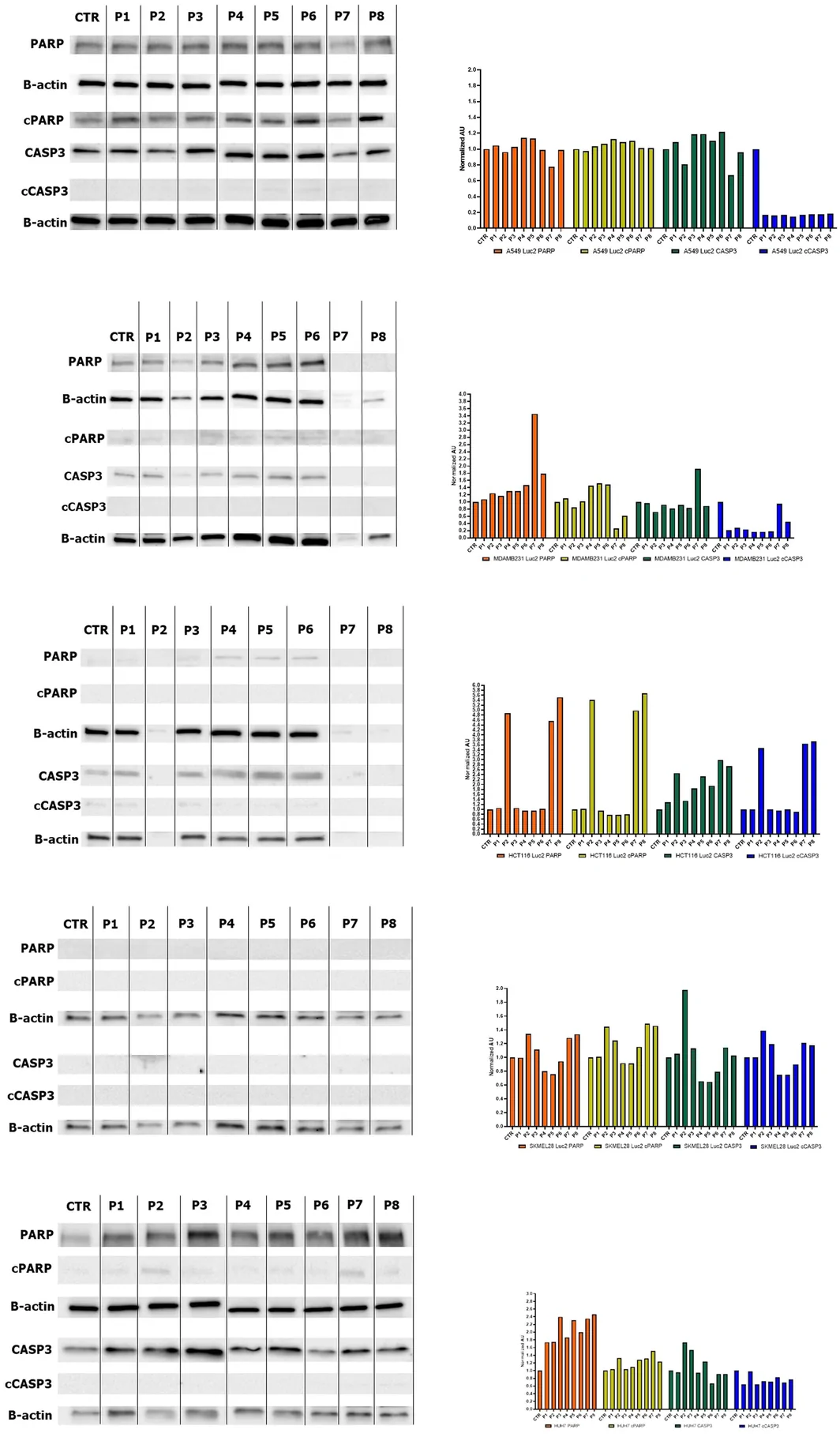
Western Blot assessment of cCaspase 3, Caspase 3, cPARP and PARP after 24 h exposure to anaesthetic compounds. Band intensity was measured using Image J and the normalization performed against each B‐Actin. The graph illustration was performed using GraphPad 8.

HUH7, the hepatocarcinoma cell line, showed various outcomes depending on the compound that was used as treatment for 24 h, all groups had increased total PARP3, with P3, P7 and P8 having the most intense signal, while cPARP was significantly increased in P2 and P7 only. In the case of CASP3 in P2, P3 and P5 groups an increase in protein amount can be observed, while the cCASP3 remains still below the control level in all groups. However, the cCASP3 was barely visible in P8 groups only, but without significant increase compared to the control. The results are presented in Figure 11.

## 4 Discussion

Modern anaesthesia has changed the approach to the patient and transformed pain control, yet its broader cellular consequences, particularly in oncology, remain incompletely understood. Anaesthetic drugs span volatile agents, intravenous agents, opioids and local anaesthetics, and accumulating evidence suggests they can modulate biomolecular pathways governing proliferation, angiogenesis, autophagy and cell death. Volatile anaesthetics (e.g., isoflurane, sevoflurane) have been reported, at clinically relevant concentrations, to upregulate HIF 1α, potentially enhancing epithelial–mesenchymal transition (EMT), angiogenesis and chemoresistance, thereby affecting survival of metastatic tumour cells. Conversely, several local anaesthetics (lidocaine, ropivacaine, bupivacaine, levobupivacaine) demonstrate antitumor actions, inducing apoptosis, lowering proliferation and modulating key signalling cascades, sometimes synergizing with chemotherapeutics, through mechanisms such as mitochondrial dysfunction, caspase activation and matrix metalloproteinase downregulation [7, 8, 54, 55, 56].

Propofol and ketamine are both intravenous agents that suppress the proliferation and migration of most tumour cell lines (lung, colon, breast, glioma, melanoma) through their dose‐dependent effects. This suppression occurs by apoptosis, arrest of the cell cycle and a reduction in the metastatic potential of these tumour cell lines. Ketamine is a proven inhibitor of proliferation and migration in lung breast and colorectal cancer xenograft models as well as inducing the formation of reactive oxygen species leading to disruption of the cell cycle progression. In contrast, opioids have been shown to have multiple heterogeneous, dose‐dependent effects on tumour biology. Fentanyl, for example, has been shown to suppress NF‐κB downregulating pro‐oncogenic transcription factors through its anti‐proliferation, invasion and migration capabilities, however there are also situations where opioids enhance stemness, epithelial‐mesenchymal transition (EMT) or angiogenesis depending on the context and concentration [19, 20, 21, 22, 25].

Not all anaesthetic compounds share anti‐tumour properties. Neuromuscular blocking agents (e.g., Rocuronium bromide, Suxamethonium) generally do not display antitumor effects in vitro, with reports of rocuronium promoting invasion and growth in certain triple negative breast cancer models and variable, cell specific effects (including growth stimulation in gastric cancer). There have been inconsistent results reported for pro‐tumour opioid effects in clinical settings, therefore it is important to continue rigorous translational research on this topic so that in vitro data can be correlated with patient outcomes [18, 31, 32].

Local anaesthetics showed increased cytotoxicity on oral cancer cells, compared to normal cells, inducing cell membrane ruptures and mitochondrial injury, promoting rather necrosis than apoptosis [16]. Lidocaine and bupivacaine showed pro‐apoptotic features against breast cancer MCF7 cell line, underscoring their potential use during breast cancer surgery, without the risk of enhancing tumour cell metastasis and proliferation [57]. Another study revealed that four local anaesthetic agents including lidocaine and ropivacaine, did not interfere with the cytostatic therapy and contribute to the tumour cell inhibition in TNBC cancer MDAMB231 cell line [12]. Human melanoma cell lines A375 and Hs294T exposed to local anaesthetic compounds for 1 h showed a reduction of viable cell population, with an increased cell death rated when increasing the exposure time up to 72 h. The study indicates the cytotoxic activity of local anaesthetic agents in time‐ concentration‐ and agent‐dependent manner [58]. Human oral squamous cell carcinoma cell lines showed sensitivity to intravenous and local anaesthetics, with an increased non‐apoptotic cell death induced by isoprenaline and dexmedetomidine [59].

In our study the cytotoxicity assessment revealed heterogenous responses of tumour cell lines to the eight anaesthetic compounds tested. Various IC_50_ values were obtained across cell types, indicating compound‐specific and cell‐specific sensitivity. Notably, noradrenaline (P1), Ropivacaine (P5), Ketamine (P7) and Propofol (P8) exhibited inhibitory effect on both tumour and normal fibroblast cells, though normal cells generally required higher concentrations for inhibition. Lidocaine (P2) showed pronounced activity against HCT116 Luc2 and HUHU7 cells, while Rocuronium Bromide (P3) and Suxamethonium Chloride (P4) had limited or negligible effects on most cell lines. Fentanyl (P6) does not demonstrate measurable cytotoxicity on any of the cell lines tested, at 48 h, probably this effect is related to the dose and exposure to fentanyl which may be different at prolonged exposure to this anaesthetic compound. The high heterogeneity in cytotoxicity effect underscores the differential susceptibility of tumour cells to anaesthetic agents, indicating that some tumour cells may be inhibited when in contact with these agents. All agents act rather as inhibitors of the cell growth, fact sustained by the mechanisms of action of anaesthetic agents. Most of them disrupt essential pathways for cell proliferation or induced stress responses rather than promoting proliferation, as in none of the tested groups the cell proliferation was stimulated, comparable with the literature [60, 61, 62].

Clinically achievable perioperative plasma concentrations represent approximate total plasma concentrations reported in adult patients receiving standard perioperative dosing regimens. Values vary according to dose, route of administration, infusion duration, sampling time, anaesthetic technique and patient characteristics. For highly protein‐bound drugs (propofol, ropivacaine and fentanyl), unbound plasma concentrations are substantially lower than total plasma concentrations. Succinylcholine chloride is characterized by an exceptionally short plasma half‐life (< 1 min), and clinically relevant concentrations are highly transient following bolus administration. Consequently, direct comparison with prolonged in vitro exposure should be interpreted cautiously.

An important consideration when interpreting the present findings is the relationship between the experimental concentrations used in vitro in our study and those achievable during routine perioperative administration.

As summarized in Table 6, clinically achievable perioperative plasma concentrations are represented within the experimental concentration ranges investigated for all agents, although the extent of representation varies among drugs. Particularly for propofol, lidocaine, ketamine, ropivacaine and fentanyl, clinically achievable perioperative plasma concentrations are encompassed within the tested concentration ranges.

**TABLE 6:** Comparison of the experimental concentration ranges used in the present study with clinically achievable perioperative plasma concentrations reported in the literature.

Compound	Experimental concentration range (present study, μM)	Clinically achievable perioperative plasma concentration (μM)	Representation of clinically achievable concentrations	Interpretation	References
Noradrenaline	0.000059–590	0.003–0.020 (up to 0.044 with high‐dose infusion)	Limited representation	The clinically achievable perioperative concentration range represents only a small fraction of the concentrations tested. Most experimental concentrations substantially exceed those encountered during routine clinical administration	[63, 64]
Lidocaine	0.000427–4270	4.27–12.80	Included	Clinically relevant perioperative plasma concentrations are represented within the tested concentration range, although the highest experimental concentrations exceed routine clinical exposure	[65, 66]
Rocuronium bromide	0.000189–1890	1.64–8.19	Included	Clinically achievable perioperative concentrations are included within the tested range, although the upper experimental concentrations greatly exceed routine clinical exposure	[67, 68]
Succinylcholine chloride (suxamethonium)	0.000554–5540	5.54–13.84 (bolus); 1.39–5.54 (continuous infusion)	Partially represented	Clinically achievable concentrations are represented within the tested range; however, exposure after bolus administration is extremely transient because of rapid plasma hydrolysis, limiting direct comparison with prolonged in vitro exposure	[69, 70]
Ropivacaine	0.000365–3650	1.82–10.20	Included	Clinically achievable perioperative plasma concentrations are represented within the tested range; however, most experimental concentrations extend well beyond those encountered during routine clinical practice	[71, 72]
Fentanyl	0.000001–14.8	0.0018–0.0446	Included	Clinically achievable perioperative plasma concentrations are represented within the tested range, although most experimental concentrations substantially exceed those encountered during routine clinical practice	[73, 74]
Ketamine	0.0021–21,000	0.42–12.62	Included	Clinically achievable perioperative plasma concentrations are well represented within the tested range, although the majority of experimental concentrations extend well beyond those encountered during routine clinical practice	[75]
Propofol	0.000561–5610	5.61–33.66	Included	Clinically achievable perioperative plasma concentrations are well represented within the tested range, although the highest experimental concentrations substantially exceed those encountered during routine clinical practice	[76, 77]

However, the concentration‐response experiments were intentionally extended well beyond clinically relevant exposure to characterize pharmacological activity and determine IC_50_ values. Consequently, biological effects observed exclusively at supraphysiological concentrations should be interpreted cautiously, as they may not be directly reproducible in vivo. Future studies integrating pharmacokinetic and pharmacodynamic data with clinically relevant exposure models are needed to better define the translational significance of these findings.

Fluorescent confocal microscopy showed profound, agent‐specific morphological alterations across all tumour cell lines. In lung cancer cells, noradrenaline (P1) and lidocaine (P2) induced mitochondrial fragmentation, nuclear condensation and F‐actin disruption, while ropivacaine (P5) and ketamine (P7) triggered stress fibre formation and osmotic swelling. Similar cytoskeleton changes were observed in colon cancer cells, where most treatments caused round cells and filament fragmentation, with P4 inducing severe nuclear damage. Hepatocarcinoma HUH7 cells displayed tunnelling nanotubes under exposure to P2, P4, P5 and P8, alongside mitochondrial depolarization in P8 group, all changes suggesting intense cellular stress. TNBC cells suffered cytoskeleton remodelling and nuclear damage, with tunnelling nanotubes in P2‐P7 groups, highlighting intracellular stress signalling. Melanoma cells, characterized by robust cytoskeleton networks, showed dramatic filament fragmentation and mitochondrial disruption under P2 and P3 treatment, while P4‐P8 stimulated the production of stress fibres and nuclear alterations.

Across all cell lines, osmotic imbalance and cell shape changes were recurrent, highlighting anaesthetic‐induced interference with cytoskeletal integrity and mitochondrial function. These findings suggest that anaesthetic compounds exert pleiotropic effects on tumour cell architecture, potentially impairing motility and viability through structural destabilization. These effects are strongly linked to the molecular analysis of cell death, cell migration and cell cycle status of the tumour cells.

The Liu et al. review synthesizes these converging mechanisms—sodium channel blockade, Ras/RhoA inhibition, cell cycle arrest, EGFR suppression, calcium influx modulation, microRNA regulation and mitochondrial dysfunction—into a unified framework for understanding how local anaesthetics affect cancer cells [78]. Furthermore, Zhang et al. [79] confirmed that lidocaine directly represses cytoskeleton formation in cancer cells, contributing to inhibition of invasion and migration through TNFα‐dependent Src/AKT/ICAM signalling. Piegeler et al. [80, 81] further demonstrated that clinically relevant concentrations of lidocaine and ropivacaine block TNFα‐induced Src‐dependent activation of focal adhesion kinase (FAK) and caveolin‐1 phosphorylation—key regulators of cytoskeletal‐membrane interactions—thereby attenuating MMP‐9 secretion and tumour cell invasion.

Flow cytometry evaluation using PoPRO1 and 7AAD staining after 24 h exposure to anaesthetic compounds revealed various effects on each cell line. In the case of A549 Luc2 cells P1, P2 and P3 induced approximatively 15% cell death, P7 and P8showed mild inhibitory effect with few apoptotic cells resulted after therapy, while P4 and P6 had no impact. In the case of MDAMB231 Luc2 Most compounds were effective, particularly P3, which triggered over 45% total apoptosis (10% early, 35% late). P6 was the only compound that did not induce significant death. For HCT116 Luc2 cells was the least sensitive; no compound exceeded a 20% death rate, with P2, P7 and P8 being the most potent. SKMEL28 Luc2 cells showed high sensitivity to P1 and P3, with 35% apoptotic cells, whereas P6 was least effective. Hepatocarcinoma cell line HUH7 showed a 20% basal apoptosis rate. Only P3 significantly increased this to 30%, representing a 50% rise over control. MDAMB231 and SKMEL28 are the most sensitive. MDAMB231 showed the most dramatic response to P3, with nearly half the population entering apoptosis. This suggests this cancer types might be the most vulnerable to these specific treatments. A549 shows a targeted sensitivity only to P1–P3, suggesting a more selective mechanism of action in lung tissue. HCT116 is the most ‘stubborn’, resisting significant death across all compounds. Similarly, HUH7 remained largely unaffected, except by P3, maintaining its basal cellular state.

Some anaesthetic compounds may have pro‐apoptotic effects (lidocaine, propofol) and they can induce an increase in cCASP3 and cPARP with a concomitant decrease in full‐length CASP3 and PARP indicates the anaesthetic is inducing caspase‐dependent apoptosis. Local anaesthetics such as lidocaine and bupivacaine have been shown to induce this pattern in breast cancer (MCF‐7), ovarian cancer (SKOV‐3), prostate cancer (PC‐3) and NSCLC (A549, H520) cell lines at clinically relevant concentrations [82, 83]. These markers typically change in a dose‐ and time‐dependent manner. PARP cleavage can be detected as early as 30–90 min after apoptotic stimulus, with DNA fragmentation trailing by approximately 30 min. The ratio of cleaved‐to‐full‐length forms (cPARP/PARP and cCASP3/CASP3) provides a semi‐quantitative measure of apoptotic intensity [84].

The apoptotic response to treatment varied significantly across cell lines, with the most notable effects observed in A549 Luc2 and HUH7 cells, where ketamine (P7) and other compounds actively modulated PARP signalling. While HCT116 cells showed a dramatic loss of protein integrity under specific treatments (P2, P7, P8), SKMEL28 melanoma cells appeared completely unresponsive via the PARP/Caspase 3 pathway, suggesting a reliance on alternative survival mechanisms. Across most lines, including MDAMB231 Luc2, total protein levels fluctuated more than their cleaved, active forms, indicating that while these treatments influence the cellular machinery, they don't always trigger full apoptotic execution within 24 h.

Hepatocellular carcinoma cells showed sensitivity to lidocaine, which arrested the cells in G0/G1 and induced apoptosis activating caspase‐3, suppressing the tumour cell development. Furthermore, cells exposed to cisplatin and received lidocaine showed better outcomes than cisplatin alone, indicating that the anaesthetic agent is promoting cell death, rather than stimulating tumour cell proliferation [85].

The gene expression included the analysis of Caspase 3 (*Casp3*), Caspase 8 (*Casp8*) and Caspase 9 (*Casp9*)– cysteine proteases central to apoptosis, Caspase 8 and Caspase 9 initiator caspases, activating downstream caspases such as Caspase 3. The three caspases are central regulators of apoptosis, Caspase 8 in the initiator of the extrinsic apoptotic pathway. Upon activation, caspase 8 cleaves and activate downstream effectors, like caspase 3 [86]. Moreover, Caspase 8 could have a non‐apoptotic effect and activate NF‐kB (*NF‐κB*) or regulate immune cell homeostasis. Caspase 9 is the initiator of the intrinsic apoptotic pathway. It is activated on the apoptosome complex following cytochrome C release from the mitochondria leading to the activation of caspase 3 and caspase 7 [87, 88].

Caspase 3 is the key apoptotic caspase, responsible for cleaving a wide array of cellular substrates to produce morphological and biochemical changes specific for apoptosis, including DNA fragmentation and membrane blebbing [89, 90]. Nrf2 (*Nrf2*), a transcription factor that regulates antioxidant response elements, is promoting expression of several genes involved in cellular defence against oxidative stress. Nrf2 can be a substrate for caspase 3 and when cleaved it could generate fragments that promote cell death, as the loss of Nrf2 function increases basal apoptosis sensitizing mitochondrial permeabilization [91, 92].

NF‐kB is a master regulator of inflammation, immune response, cell survival and is activated by various stimuli including caspase 8, and has critical roles in cancer, autoimmune diseases and inflammation. Opioids activate NF‐κB through both μ‐opioid receptor (MOR)‐dependent and TLR4‐dependent mechanisms. MOR activation induces NF‐κB via PKCζ‐mediated p65 phosphorylation, driving proinflammatory chemokine expression (CCL2, CCL5, CXCL10). Morphine potentiates LPS‐induced NF‐κB in microglia through PKCε signalling, promoting a proinflammatory phenotype. In the cancer context, NF‐κB activation promotes tumour cell proliferation, survival, EMT‐mediated invasion, therapy resistance and angiogenesis [93, 94]. Furthermore, local anaesthetics attenuate inflammatory signalling upstream of NF‐κB by inhibiting Src‐dependent activation of Akt and FAK, suppressing TNFα‐induced inflammatory cascades. They also induce ER stress (PERK/eIF2α/ATF4 axis), autophagy, and immunogenic cell death markers (ATP and HMGB1 release), which represent stress responses that can either feed into or counteract NF‐κB‐driven survival signalling depending on the cellular context [60, 80, 81].

NF‐kB is a central transcription factor in inflammation, cell survival and cell cycle regulation, promoting cell cycle progression by upregulating Cyclin D1, facilitating CDK3‐cyclin D activity and G1/S transition. Moreover, NF‐kB suppresses apoptosis by inducing anti‐apoptotic signals, inhibiting particularly caspase 8 and caspase 3 [95, 96]. CDK2‐Cyclin A (*CDK2CYA*), CDK1‐Cyclin (*CDK1CY*) and CDK3‐Cyclin D (*CDK3CYD*) regulate cell cycle progression, CDK4CYD controls G1 phase, CDK2CYA regulates S phase and CDK1CY control G2/M transition—this cyclin activity is a hallmark of many tumours [89, 97]. CDK2‐cyclin A becomes active in late S and G2 phases, promoting DNA replication and preparation for mitosis, it phosphorylates components of the DNA replication machinery and coordinates nuclear events for proper timing of mitotic entry [98, 99]. CDK1‐cyclin complexes are essential for G2/M transition and mitosis, CDK1 is strictly required for cell cycle completion in mammals, and its activation triggers chromosome condensation, nuclear envelope breakdown and mitotic spindle formation. Moreover, CDK1 can compensate for loss of other interphase CDKs by associating with multiple cyclins underscoring its central role [100, 101]. CDK4‐cyclin D is active in early G1 phase, phosphorylating retinoblastoma protein (Rb) to release E2F transcription factors, promoting entry into S phase, and DNA synthesis [102, 103, 104].

In lung cancer cell line A549 Luc2, all caspases had slight changes in terms of gene expression, however, *Caspase 8* and *Caspase 9* display an ascendent trend without a statistically significant change observed. No significant changes are observed in *NRF2* or cyclins, while the only significant change observed in this cell line is at *NF‐kB* in the group treated with lidocaine compared to control, where a slight upregulation is visible (*p* = 0.031). This suggests that the cell line has a response to treatment even at 48 h, indicating that a prolonged exposure to the anaesthetic compounds may offer different perspectives in terms of gene expression changes post‐treatment.

The colon cancer cell line HCT116 Luc2 had a better outcome with multiple statistically significant changes observed. All caspase genes were upregulated in groups treated with P5, P6, P7 and P8, with *Caspase 8* showing the most visible changes. Similar trend, but without statistically significant output was observed in most of the groups for cyclins, only in the case of CDK2CYA for P8, the upregulation was statistically significant (*p* = 0.048). However, in this case, for cell treated with P3, P4 and P5, a significant downregulation was observed in *NRF2* gene, indicating a loss of redox homeostasis in these cells [105, 106].

Hepatic cancer cell line HUH7, had no significant changes displayed in caspases, *NRF2* and *NF‐kB*, while the *CDK1CY* gene was upregulated in all treatments, with P4, P5 and P7 showing statistical significance. On the other hand, CDK4CYD gene was downregulated in all groups, with P4 having significant downregulation (*p* = 0.035). These changes suggest that there is a shift in the cell cycle which may interfere with cell proliferation. CDK4‐cyclin D is essential for progression through G1 phase, and an inhibition of the gene is usually translated into a cell cycle arrest in G1 and is associated with cytotoxicity and tumour suppression [107]. The upregulation of CDK1‐cyclin B gene promotes entry into G2/M, promoting cell growth, however in this case, it may indicate that tumour cells try to compensate for the G1 cell cycle arrest and try to shift into G2/M phase [108], with evidence in the cell cycle analysis, where the G2/M phase is increased compared to the control in several groups. However, it is of high importance to mention that HUH7 cells have a baseline of G2/M phase already increased, compared to other cell lines used, and we may consider the changes based on this initial status.

In the case of TNBC cell line MDAMB231 Luc2, a coordinated cellular response is observed, as the decreased viability is linked to the state of the necrosis with both caspase dependent and independent mechanisms involved. The increased gene expression of caspases is a result of cytotoxic stress, with visible differences in P2 and P8 treated groups. G1 phase accumulation and decreased G2/M phase suggest cell cycle arrest in G1 phase, being associated with downregulation of CDK4‐cyclind D gene [109]. The upregulation of CDK1‐cyclin and CDK2‐cyclin A genes are related to a compensatory mechanism of dysregulated attempt to drive cell cycle. Increased *NRF2* is an indicator of adaptive response to oxidative stress, induced by the treatment, and P2 and P8 displayed the most significant upregulation of the gene, however due to the overwhelming toxicity and cell death initiation, its upregulation may not be sufficient to overcome the effects. This pattern of an interplay between cell cycle regulation, cell death and oxidative stress adaptation can highlight a successful anti‐tumour effect on specific groups.

SKMEL28 Luc2 melanoma cells had shift towards Late apoptosis/necrosis in all treated groups, with the most visible effect in the group treated with P1, and all the cells shifted to G2/M phase, due to cellular stress or DNA damage which sensitizes the cells to undergo necrosis. The activation of G2/M cell cycle arrest and necrosis, with partial caspase 8 activation and NRF2 pathways and no significant modulation of cyclins genes, mostly in P1 traded group, is indicating that all anaesthetic compounds, notably noradrenaline induce cell death and highlights an interplay between adrenergic signalling, cell cycle regulation and oxidative stress adaptation [110, 111].

The main limitation of our study is represented by the in vitro experimental model which does not simulate the complexity of the tumour in real life setting. The effects that were observed are due to anaesthetic agents' interaction with tumour cells only, without the modulation induced by the immune system and tumour microenvironment.

## 5 Conclusions

The anaesthetic compounds tested here exhibited heterogeneous, cell‐line‐specific effects on cell viability, migration, colony formation and gene expression after 24 and 48 h treatment, with most compounds acting as growth inhibitors and anti‐tumoral agents rather than stimulators. While certain compounds such as lidocaine and propofol demonstrated significant cytotoxicity and modulation of cell death pathways, others showed minimal impact, underscoring the complexity of anaesthetic agent–tumour interactions. However, these findings must be interpreted within the inherent limitations of two‐dimensional monolayer culture systems, which lack the stromal architecture, immune cell populations, extracellular matrix components, and dynamic pharmacokinetic conditions that characterize the in vivo tumour microenvironment.

Notably, the perioperative setting involves concurrent neuroendocrine stress responses, transient immunosuppression and complex immune–tumour crosstalk—biological variables that cannot be recapitulated in isolated cell systems and that may substantially modify the net effect of anaesthetic agents on tumour biology.

Furthermore, the drug concentrations and sustained exposure times employed in vitro often exceed those achievable during clinical anaesthesia, and available randomized clinical trials have largely demonstrated neutral effects of anaesthetic technique on long‐term oncologic outcomes.

These findings suggest that several anaesthetic agents may exert concentration‐dependent effects on cancer cell biology. However, the translational significance of these observations depends on whether the effective concentrations are achievable during routine perioperative administration and therefore requires confirmation in appropriate in vivo and clinical studies.

## Author Contributions

**Andrei Ivancuta:** methodology, formal analysis. **Olga Grajdieru:** conceptualization, software, writing – original draft. **Adrian Bogdan Tigu:** conceptualization, methodology, software, formal analysis, resources, data curation, writing – original draft, writing – review and editing, visualization, funding acquisition. **Ximena‐Maria Muresan:** methodology, formal analysis, writing – review and editing. **Cristian Silviu Moldovan:** methodology, formal analysis, writing – original draft, visualization. **Alexandru Cristian Sabo:** conceptualization, methodology, software. **Andrada‐Ioana Balmez:** methodology. **Andrada Uhl:** methodology, formal analysis. **Catalin Constantinescu:** investigation, validation, resources, project administration, supervision. **Simina Pîrv:** methodology. **Miruna Cristina Ungurenasu:** methodology. **Madalina Nistor:** methodology, formal analysis, writing – original draft. **Ada Moisescu:** methodology. **Diana Cenariu:** writing – review and editing, methodology, formal analysis. **Ciprian Tomuleasa:** validation, data curation, investigation, project administration, resources, supervision.

## Funding

C.C. was financed from an internal grant of Iuliu Hațieganu University (32154/25/16.12.2024) and Young Research Teams 2023: (PN‐IV‐P2‐2.1‐TE‐2023‐0483).

## Ethics Statement

The authors have nothing to report.

## Consent

The authors have nothing to report.

## Conflicts of Interest

The authors declare no conflicts of interest.

## Data Availability

The data that support the findings of this study are available from the corresponding author upon reasonable request.
